# Is TCR/pMHC Affinity a Good Estimate of the T-cell Response? An Answer Based on Predictions From 12 Phenotypic Models

**DOI:** 10.3389/fimmu.2019.00349

**Published:** 2019-03-04

**Authors:** Jesús Gálvez, Juan J. Gálvez, Pilar García-Peñarrubia

**Affiliations:** ^1^Department of Physical Chemistry, Faculty of Chemistry, University of Murcia, Murcia, Spain; ^2^Department of Computer Science, University of Illinois at Urbana-Champaign, Urbana, IL, United States; ^3^Department of Biochemistry and Molecular Biology B and Immunology, School of Medicine, University of Murcia, Murcia, Spain

**Keywords:** TCR-pMHC interaction, affinity, correlation between affinity immune response, phenotypic models, T-cell activation

## Abstract

On the T-cell surface the TCR is the only molecule that senses antigen, and the engagement of TCR with its specific antigenic peptide (agonist)/MHC complex (pMHC) is determined by the biochemical parameters of the TCR-pMHC interaction. This interaction is the keystone of the adaptive immune response by triggering intracellular signaling pathways that induce the expression of genes required for T cell-mediated effector functions, such as T cell proliferation, cytokine secretion and cytotoxicity. To study the TCR-pMHC interaction one of its properties most extensively analyzed has been TCR-pMHC affinity. However, and despite of intensive experimental research, the results obtained are far from conclusive. Here, to determine if TCR-pMHC affinity is a reliable parameter to characterize T-cell responses, a systematic study has been performed based on the predictions of 12 phenotypic models. This approach has the advantage that allow us to study the response of a given system as a function of only those parameters in which we are interested while other system parameters remain constant. A little surprising, only the simple occupancy model predicts a direct relationship between affinity and response so that an increase in affinity always leads to larger responses. Conversely, in the others more elaborate models this clear situation does not occur, i.e., that a general positive correlation between affinity and immune response does not exist. This is mainly because affinity values are given by the quotient *k*_on_/*k*_off_ where *k*_on_ and *k*_off_ are the rate constants of the binding process (i.e., affinity is in fact the quotient of two parameters), so that different sets of these rate constants can give the same value of affinity. However, except in the occupancy model, the predicted T-cell responses depend on the individual values of *k*_on_ and *k*_off_ rather than on their quotient *k*_on_/*k*_off_. This allows: a) that systems with the same affinity can show quite different responses; and b) that systems with low affinity may exhibit larger responses than systems with higher affinities. This would make affinity a poor estimate of T-cell responses and, as a result, data correlations between affinity and immune response should be interpreted and used with caution.

## 1. Introduction

TCR-pMHC interaction leading to T cell activation is the keystone of the adaptive immune responses against infections and cancer, and plays a decisive role in allergy, autoimmunity and transplant rejection (1). The engagement of TCR with its specific antigenic peptide (agonist)/MHC complex (pMHC) triggers intracellular signaling pathways that induce the expression of genes required for T cell-mediated effector functions, such as T cell proliferation, cytokine secretion and cytotoxicity (2). The underlying mechanisms for these unique features of T cells function remain enigmatic, and different hypothesis, verbal and theoretical models have been proposed along the past decades to explain T cell activation [reviewed by (3, 4)]. Nevertheless, the outcome of a T-cell response must be determined by the biochemical parameters of the TCR-pMHC interaction since on the T-cell surface the TCR is the only molecule that senses antigen. Of these parameters, one of the most extensively studied has been the TCR-pMHC affinity (5, 6), based on the current assumption that the highest affinity T cells have a competitive advantage during the immune response because they would receive stronger and more prolonged activation signals that T cells with lower affinity interactions (7–13). However, and despite extensive experimental work, it has not been possible to establish a clear correlation pattern between affinity and T-cell response because the available data are far from conclusive and even contradictory (4, 14–33). Here, we perform a detailed study of the relationship between affinity and TCR-pMHC interaction based on the following assumptions: (a) T-cell response triggered by TCR-pMHC interaction is a very complex process which, in addition to the binding rate constants, could be influenced by the length and kinetics of the activation chain, negative feedback, limited and sustained signaling, and antigen doses, among others i.e., is a multiparameter process; (b) to determine without ambiguity the influence exerted by a given parameter we should analyze the response by varying only this parameter while the other ones are kept constant. However, affinity is an intrinsic property of the effector-target system that cannot be modified without altering other properties of the system, i.e., if somehow affinity is changed we cannot guarantee that other system parameters have remained unchanged; (c) we could compare T-cell responses of systems with different affinities. But again, we cannot guarantee that systems that differ in affinity have the same values for the remaining parameters; (d) furthermore, affinity used as an estimate of immune responses is questionable since its values are given by the quotient *k*_on_/*k*_off_ (where *k*_on_ and *k*_off_ are the rate constants of the binding process), and different sets of these rate constants can give the same value of affinity. In other words, that affinity is in fact not a parameter but a quotient of two parameters (*k*_on_ and *k*_off_) which can be involved, independently of affinity and of each other, in the T-cell response. Under these conditions there could be systems with different values of *k*_on_ and *k*_off_ (but the same value of affinity) that give different responses. And even, that systems with lower affinity exhibit larger responses than systems with higher affinities. Obviously, this would make affinity a poor estimate to evaluate T-cell responses, and thus, data correlations between affinity and immune response should be interpreted and used with caution.

These complications make experimentally difficult to assess without uncertainty the relationship between affinity and immune response and, for this reason, other approaches to the problem seem more appropriate. Currently, there is great research effort in developing TCR-based immunotherapies by increasing TCR affinity to improve the therapeutic effect of TCR gene-modified T-cells in cancer patients. Nevertheless, several clinical trials using high affinity TCRs in adoptive cell transfer have reported unexpected and severe adverse effects, such as death and off-target cross-reactivity. Those results point out that less emphasis need to be placed on TCR-pMHC affinity as a means of predicting or increasing the therapeutic effectiveness of TCR gene-modified T-cells used in adoptive cell transfer. Hence, a better understanding of antigen recognition and T-cell activation is necessary to improve the treatment efficacy and safety in cancer patients (29, 30, 34, 35). In this context, a useful approach is the use of phenotypic models which have shown very promising in describing the main characteristics of the T-cell response (4, 14, 23, 36–38). In addition, they have the advantage that are not restricted by the above limitations since allow us to study the response of a given system as a function of only those parameters in which we are interested while other system parameters remain constant. Taking into account the above considerations, we have determined the relationship between TCR-pMHC affinity and T-cell response based on the predictions from 12 phenotypic models and proceeding as described in the following sections.

## 2. Materials and Methods

### 2.1. Phenotypic Models

The influence exerted by the TCR-pMHC affinity on the immune response was studied by performing computations from 12 phenotypic models with the specific aim of analyzing the relationship between affinity and response. To this end, the following phenotypic models were considered:

- occupancy
- kinetic proofreading (kpr)
- kpr with limited signaling
- kpr with sustained signaling
- kpr with negative feedback
- kpr with induced rebinding
- kpr with stabilizing activation chain
- kpr with limited and sustained signaling
- kpr with negative feedback and limited signaling
- kpr with stabilizing activation chain and limited signaling
- kpr with stabilizing activation chain and sustained signaling
- kpr with limited signaling coupled to an incoherent feed-forward loop (kpl-iff)

Models **(a)-(d)** are reviewed in Lever et al. (4), model **(e)** is described in Lever et al. (4) and François et al. (38), model **(f)** in Dushek and van der Merwe (39), model **(g)** in Gálvez et al. (37), and model **(l)** in Lever et al. (23). In addition, and because the modular structure of phenotypic models allows to study the effect exerted on the response by adding assumptions and new parameters to a simpler model, we have formulated the new models **(h)-(k)** by combining hypothesis of models **(c)** and **(d)**, **(c)** and **(e)**, **(g)** and **(c)**, and **(g)** and **(d)**, respectively. Thus, for example, in developing model **(h)** (kpr with limited and sustained signaling), we have considered that assumptions for limited signaling [namely, that TCRs having reached the signaling competent state can only signal for a limited period because of movement into the immunological synapse or to become tagged for removal (4)] and those for sustained signaling [i.e., that signaling competent TCRs are able to maintain signaling for a prescribed period of time, even after pMHC unbinding (4)] could be involved together in determining the T-cell response. Schemes of these models and of the corresponding parameters are shown in Figures 1A, B.

**Figure 1A:**
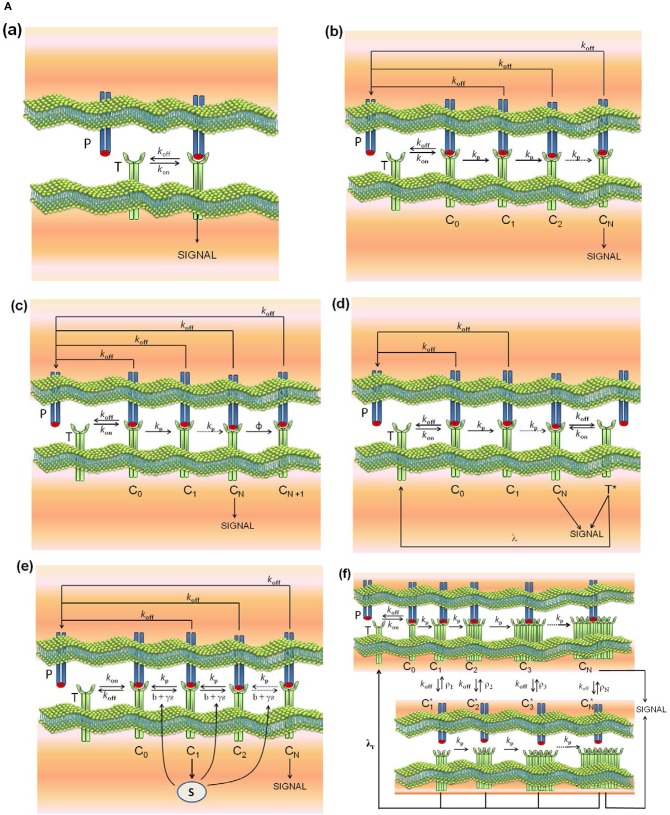
Schemes of the phenotypic models. Panel models: **(a)** occupancy; **(b)** basic kpr; **(c)** kpr with limited signaling; **(d)** kpr with sustained signaling; **(e)** kpr with negative feedback; **(f)** kpr with induced rebinding. A full description of these models are found in the references given in main text.

**Figure 1B:**
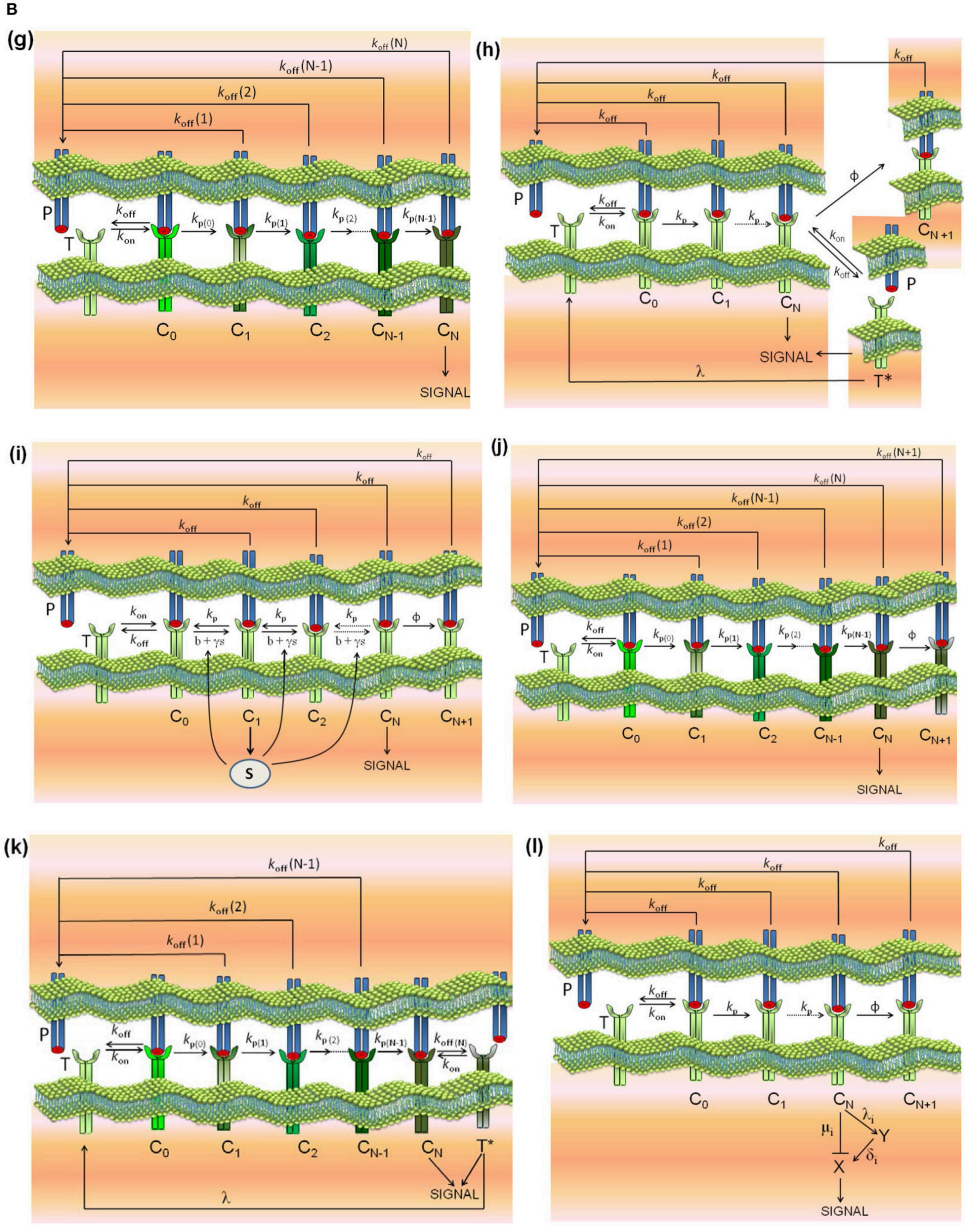
Schemes of the phenotypic models. Panel models: **(g)** kpr with stabilizing activation chain; **(h)** kpr with limited and sustained signaling; **(i)** kpr with negative feedback and limited signaling; **(j)** kpr with stabilizing activation chain and limited signaling; **(k)** kpr with stabilizing activation chain and sustained signaling; **(l)** kpr with limited signaling coupled to an incoherent feed-forward loop (kpl-iff). A full description of these models are found in the references given in main text.

### 2.2. Parameter Values

All models have a common set of parameters. Also, there are parameters specific to each model. The common set of parameter values used for computation in this work are similar to those used in Lever et al. (4), Altan-Bonnet et al. (14), Gálvez et al. (37), and François et al. (38): number of TCRs, $T_{T}=2\times 10^{4}$; $k_{\text{on}}=5\times 10^{-5}s^{-1}$; $k_{p}=1s^{-1}$. There is no concentrations units: all concentrations in figures, tables and in rate constants are per cell. Thus, $k_{\text{on}}=5\times 10^{-5}{\left(molecule.s\right)}^{-1}$ and *k*_off_ = 1/τ where τ is the dissociation time of the TCR-pMHC complex. Besides the common parameters, models **(c)-(l)** also include specific parameters that are defined in Figures 1A, B. Their numerical values are listed next and are similar to those used in the accompanying references:

- model **(c)**, kpr with limited signaling (4): ϕ = 0.09*s*^−1^. This value of ϕ was used in all models including limited signaling [models **(c)**, **(h)**, **(i)** and **(l)**].
- model **(d)**, kpr with sustained signaling (4, 40): λ = 0.001*s*^−1^. This value of λ was used in all models including sustained signaling: **(d)**, **(h)**, and **(k)**.
- model **(e)**, kpr with negative feedback (4, 38): $S_{T}=6\times 10^{5}$, $C_{S}=5\times 10^{3}$, β = 1*s*^−1^, α = 2 × 10^−4^*s*^−1^, *b* = 0.04*s*^−1^, γ = 4.4 × 10^−4^*s*^−1^.
- model **(f)**, kpr with induced rebinding (39): $\rho_{i}=10^{3}s^{-1}$ for *i* ≤ 21 increasing to 10^7^*s*^−1^ for *i* = 25, $\lambda_{r}=10^{4}s^{-1}$.
- model **(g)**, kpr with stabilizing activation chain (37): *r* = 1.5 in equation for *k*_off_(*i*), and *r* = 1.03 in equation for *k*_*p*_(*i*) [Equations 8, 11 in (37)].
- model **(h)**, kpr with limited and sustained signaling: ϕ = 0.09*s*^−1^, λ = 0.001*s*^−1^ [see models **(c)** and **(d)**].
- model **(i)**, kpr with negative feedback and limited signaling: ϕ = 0.09*s*^−1^, β = 1*s*^−1^, $S_{T}=6\times 10^{5}$, $C_{S}=5\times 10^{3}$, α = 2 × 10^−4^*s*^−1^, *b* = 0.04*s*^−1^ [see models **(e)** and **(c)**].
- model **(j)**, kpr with stabilizing activation chain and limited signaling: ϕ = 0.09*s*^−1^, *r* = 1.5 (*k*_off_(*i*)), *r* = 1.03 (*k*_*p*_(*i*)) [see models **(c)** and **(g)**].
- model (k), kpr with stabilizing activation chain and sustained signaling: λ = 0.001*s*^−1^, *r* = 1.5 (*k*_off_(*i*)), *r* = 1.03 (*k*_*p*_(*i*)) [see models **(d)** and **(g)**].
- model **(l)**, kpr with limited signaling coupled to an incoherent feed-forward loop (kpl-iff) (23): *Y*_*T*_ = 100, *X*_*T*_ = 100, $\gamma_{+}^{y}=1s^{-1}$, $\gamma_{-}^{y}=500s^{-1}$, $\gamma_{+}^{x}=1s^{-1}$, $\gamma_{-}^{x}=500s^{-1}$, $\lambda_{i}=0.5s^{-1}$, $\delta_{i}=50s^{-1}$, $\mu_{i}=2.5s^{-1}$.

In turn, the number of steps leading to the productive signaling complex (this parameter does not apply for the occupancy model) was *N* = 10 for all models except in the kpr with induced rebinding model where *N* = 25 [due to the assumed values of the rebinding rate constants (39), induced rebinding has little effect on the response when *N* < 25 (37, 39) and thus, if plots in this work are computed with *N* = 10 the resulting *R*-values are very close to those obtained with the basic kpr model].

Finally, it is worth to note that recent works have placed an added emphasis on experimental measurements of 2D *k*_on_ and *k*_off_ reaction rate constants vs. the corresponding 3D values (3, 41–44). Thus, while 3D TCR-pMHC interactions have been widely studied by surface plasmon resonance (SPR) (3, 44), determination of the 2D binding parameters is much more challenging and more elaborate techniques, such as adhesion frequency and thermal fluctuation assays (41), single-molecule microscopy and fluorescence resonance energy transfer (FRET) between fluorescently tagged TCRs and their cognate pMHC ligands (42), and a laminar flow chamber to monitor at the single molecule level the 2D TCR-pMHC interactions (43), were used. For computation purposes we have to take into account that 2D on-rates are actually effective on-rates with different units to the corresponding 3D on-rates which precludes that a direct comparison between 2D and 3D on-rates can be performed. Conversely, 2D and 3D off-rates have the same units (*s*^−1^), and a direct comparison is possible. Nevertheless, in Zarnitsyna and Zhu (3) we have that 2D on-rates span a broad 4-log range while 3D on-rates are compressed into a narrow range (see Figure 3B in that reference). The set of on-rates used for computation in this work spanned both narrow and broad log ranges (from 0 to 4) so that the full range of 2D and 3D on-rates values is included. As regards the off-rates, Figure 3C in Zarnitsyna and Zhu (3) shows that the 2D *k*_off_-values span approximately between 2 and 10 *s*^−1^ while this range for the corresponding 3D values is from 0.001 to 0.1*s*^−1^. The values of *k*_off_ used for computation in this work are between 0.0001 and 10*s*^−1^ so that the full range of 2D and 3D off-rates is also covered.

### 2.3. Computations and Numerical Solution of the System of ODEs

All models in Figures 1A, B were described by systems of ordinary differential equations (ODEs). Numerical solutions of the system of ODEs as a function of time, as well as all remaining calculations and plots were performed using *Mathematica 11.2*. The system of ODEs for the above models are given in Appendix and in Supplementary Material.

Responses were computed both in transient phase as a function of the activation time and in steady-state. For most models, analytical steady-state solutions can be derived, although some of them are quite complicated and must be solved numerically. For this reason, responses were always computed in transient phase from *t* = 0 until values of *t* sufficiently large. Under these conditions a steady-state for all models was reached, which was confirmed by testing that the responses obtained from transient phase solutions at *t*>>1 gave the same values than those computed from analytical (or numerical) steady-state solutions. This procedure has the advantage that, in addition to show the behavior of the response at longer times (steady-state solution), its characteristics at shorter times (transient phase solution) can be also studied.

Since a set of common parameters is used, not only qualitative but also quantitative responses from different models can be compared. This also allows us to reveal the influence exerted on the response by those parameters which are specific for each model. To this end, non-normalized responses are always provided instead of normalized values because for comparative purposes normalized responses could be misleading or even meaningless if the normalization factors are different. On the other hand, the modular structure of phenotypic models allows to show the effect exerted on the response by the new assumptions and parameters added to a simpler model. For example, we shall find that responses in models including sustained signaling are much larger, but the time required to reach the steady state is also much longer. For computational purposes it is interesting to note that solving the system of ODEs as a function of time always gives a single solution (which is the correct one) for all phenotypic models. However, for models including negative feedback [models **(e)** and **(i)**] the steady-state solutions are obtained by solving a polynomial equation with several solutions of which only one is a valid value. To determine the correct value the safest way is to compare the polynomial solutions with the transient phase values at *t*>>1. Finally, it is worthy of mention that in those models including limited signaling (see below), conclusions drawn from quantitative analysis of *R*-values at short times and under steady-state conditions (*t*>>1) can be different. In this regard, it has been suggested (45, 46) that time-scales in T-cell activation can be more relevant than responses obtained under steady-state where the *R*-values are time-independent (this should lead to questioning why then T-cell responses are normally measured and analyzed in steady-state although, more likely, is due to that experimental measurements and theoretical solutions in steady-state are much easier to deal with than those in transient phase).

### 2.4. Characteristics of the Models and Mathematical Assumptions

This section provides a brief description of the phenotypic models **(a)-(l)** used in this work, their mathematical assumptions, and their levels of complexity, bearing in mind that models **(h)-(k)** are new models which have not been previously described in the literature. The reader interested in more details on the problems discussed in this section can consult the review articles (3, 4, 47, 48) and references (14, 49–52). Also, the following considerations should be taken into account: (a) in general, quantitative predictions from a given model rely on the premises on which the model is built. Thus, in the case of TCR-pMHC interaction if a model is aimed at describing the binding process, its quantitative predictions are necessarily limited to the formation of the TCR-pMHC complex. Hence, predictions from this type of models that go beyond the binding process, v.g. on the length and kinetics of the activation chain, negative feedback, rebinding, and limited and sustained signaling, among others, would be only assumptions since such predictions can not be quantified by computation; (b) any model aimed at describing quantitatively T-cell responses should be able to consider and quantify the following key features of this response: its impressive capacity of speed, sensitivity and discrimination that allows to detect foreign pMHCs at very low concentration among much more abundant self-pMHC ligands (37–39); (c) recent works have placed an added emphasis on experimental measurements of 2D *k*_on_ and *k*_off_ reaction rate constants, comparison with the corresponding 3D values, and suggestions of how these 2D rate constants influence the T-cell response (3, 41–44). Unfortunately, a complete and rigorous theoretical integration of 2D and 3D domains in cell signaling processes is at present an extremely problem to tackle because we have to develop spatio-temporal models involving partial differential equations, deal with the complex problem of “reduction of dimensionality” (3D → 2D → 3D), and consider mass transport processes that show trafficking dynamics of ligands, cell surface components, intracellular signaling molecules through the different domains and interfaces (2D and 3D) of the system, among others (52). In turn, this means that a much larger set of kinetic parameters (most of which are unknown) are involved. Because of these difficulties phenotypic models are developed that, although they do not consider in detail all signaling events and spatial domains, they can provide a reasonable overview of the overall process on the basis of a minimum set of assumptions and parameters. This has been the approach followed in the present manuscript.

**(a)** occupancy model: is the basic model for TCR-pMHC binding and T-cell response (Figure 1A, panel a). The model is based on assuming that T-cell activation is proportional to the number of TCR-pMHC complexes formed in the binding process. Its mathematical solution is very simple (see Appendix) because the process involves only one step with only two parameters *k*_on_ and *k*_off_. Also, it is the only model that predicts a direct positive correlation between affinity and T-cell response. This model has been ruled out by its poor discrimination capacity and by experiments in which an increase in pMHC concentration with low dissociation time failed to yield the expected outcomes of activating T cells (4, 39). Transient phase values are obtained numerically, although in steady-state an analytical solution can be easily derived.
**(b)** kinetic proofreading (kpr) (53): this model assumes that TCR-pMHC interaction needs to take place during a minimum time to cause T-cell activation. In this mechanism pMHC ligands bind to TCRs to form a TCR-pMHC complex (*C*_0_) which goes through a sequence of N biochemical modifications (complexes *C*_1_, …, *C*_*N*_) which form the proofreading or activation chain until a signaling-competent state (*C*_*N*_) is attained (Figure 1A, panel b). Since in this chain only *C*_*N*_ is the productive signaling complex it introduces a delay in the activation transmission that must fulfill with the minimum threshold time required for successful signaling. Transient phase values must be obtained numerically as described in Appendix, although in steady-state an analytical solution can be derived (4, 53). The activation chain improves dramatically discrimination between pMHC ligands by amplifying small differences of their dissociation times from the TCR-pMHC complexes. However, this increase of specificity occurs by reducing the sensitivity (37–39), and to overcome this difficulty more elaborate models have been incorporated in the kpr scheme.
**(c)** kpr with limited signaling (4): this model extends the kpr mechanism by assuming that TCRs that have reached the signaling-competent state (the *C*_*N*_ complex) only signal for a limited period of time (Figure 1A, panel c). This has been ascribed to the fact “that TCR signaling is limited to the transit of TCRs from the periphery to the center of the immunological synapse and/or that the TCRs cease to signal once they are tagged for removal from the T cell surface” (4). The authors claim that this model is most compatible with experimental data, although to the best of our knowledge, its capacity of discrimination, sensitivity and speed has not been quantified. As in the kpr model, transient phase values are obtained numerically, although an analytical solution for the steady-state exists (4).
**(d)** kpr with sustained signaling (4, 16): this model is also an extension of the kpr scheme (Figure 1A, panel d) and incorporates, as suggested by some experimental data (16, 40), that signaling competent TCRs are able to maintain signaling for a prescribed period of time, even after pMHC unbinding. Due to this fact this model provides the largest values of *R* than any of the other models discussed in this work (see below). Further details, and inconsistencies of the model predictions with experimental results are discussed in review (4). As in previous models, the response as a function of time must be computed numerically, although values of the response in steady-state can be obtained from an analytical solution (4).
**(e)** kpr with negative feedback (38): this model extends the kpr scheme by considering that the rate of the complexes in the activation chain can be adjusted at intermediate stages and/or in the final signaling state *C*_*N*_ (Figure 1A, panel e). This is accomplished through a single negative feedback mediated by the Src homology 2 domain phosphatase-1 (SHP-1). Complete details are found in the original publication (38), and a overview of its predictions in review (4). Responses in transient phase and under steady-state conditions (*t*>>1) are better computed by solving numerically the system of ODEs (see Appendix) because a single solution (which is the correct one) is always obtained. Analytical steady-state solutions can be also obtained but in this case it is necessary to solve a polynomial equation with several solutions of which only one is a valid value.
**(f)** kpr with induced rebinding (39): it is a modification of the standard kpr model to allow for pMHC rebinding (Figure 1A, panel f). This model was proposed to enhance the sensitivity of the basic kpr model while retaining specificity and it was ascribed to processes “such as TCR clustering, conformational changes, and/or membrane alignment". However, the results obtained depend strongly on the assumed values for the rebinding rate constants and the value of N in the activation chain (37, 39). The responses in transient phase and in steady-state are obtained by solving numerically the corresponding system of ODEs (see Appendix). No analytical solution under steady-state conditions has been provided.
**(g)** kpr with stabilizing activation chain (37): this model (Figure 1B, panel g) is based on the assumption that the activation proofreading chain behaves differently for foreign and self pMHCs so that the complexes responsible for T cell activation stabilize (for foreign peptides), or weaken (for self-pMCH ligands), resulting in a dramatic increase in sensitivity and specificity that fulfill the criteria b) above mentioned. Stabilization and destabilization of complexes may be caused by conformational changes (54, 55), rebinding, or any other process leading to variations in the dissociation rate constants of the complexes transmitting the activation. The activation chain speeds up and larger increases in sensitivity and discrimination are enhanced even more if the rate of activation along the proofreading chain increases for foreign pMHCs and decreases for self ligands. The numerical solution for the transient phase and the analytical expression for the steady state response as a function of *k*_off_(*i*)(*i* = 0, 1, …, *N*) are shown in Appendix.
**(h-k)** The models (h)-(k) (Figure 1B, panels h–k) have been built taking into account the modular structure of phenotypic models that allows to study the effect exerted on the response by adding assumptions and new parameters to a simpler model. Thus, we have formulated the new models **(h)-(k)** by combining hypothesis of models **(c)** and **(d)**, **(c)** and **(e)**, **(g)** and **(c)**, and **(g)** and **(d)**, respectively. Transient phase solutions are obtained by solving numerically the corresponding systems of ODEs (see Appendix) and for model (j) (kpr with stabilizing activation chain and limited signaling) an analytical solution in steady-state is also given.
**(l)** kpr with limited signaling coupled to an incoherent feed-forward loop (23): this model is an extension of model **(c)** (kinetic proofreading with limited signaling model) and it was developed to take into account dependence of the T-cell response on the antigen affinity/dose. To our knowledge the model has not been quantitatively tested regarding the fulfilment of the criteria of speed, sensitivity and discrimination. The transient phase response is computed by solving numerically the corresponding system of ODEs while under steady-state conditions an analytical solution can be derived (see Appendix).

Summarizing: the developing of these models show that to take into account a new effect we need to figure out how to incorporate this effect (and the kinetic steps in which it is involved) in the mathematical framework of the phenotypic model. Thus, the response, *R*, in each model refers to the assumptions and parameters on which the model is built. For example, the occupancy model only considers the first binding step, while in the basic kinetic proofreading model *R* takes into account both the first binding step and the activation proofreading chain.

## 3. Results and Discussion

To assess whether binding affinity is a reliable estimate of T-cell responses we have determined from all models the influence exerted by antigen affinity on the corresponding responses in four different types of TCR-pMHC systems: 1) systems with the same affinity but different values of *k*_on_ and *k*_off_; 2) systems with different affinities and different values of *k*_on_ and *k*_off_; 3) systems with different affinities but the same value of *k*_off_; and 4) systems with different affinities but the same value of *k*_on_.

### 3.1. Systems With Equal Affinity but Different Values of *k*_on_ and *k*_off_

TCR-pMHC affinity is given by *A* = *k*_on_/*k*_off_. Hence, the values of *k*_on_ and *k*_off_ should change proportionally so that affinity remains constant. In Figures 2A, B we have plotted the predicted T-cell responses given by *R* (see Appendix) computed from the 12 phenotypic models for three different systems with equal affinity and the following values of *k*_on_ and *k*_off_:

**Table:** 

**System**	***k***_on_(s^−1^)	***k***_off_(s^−1^)	**τ (s)**	***A***	**Color curves in Figures 2A, B**
1	*k*_on_	0.01	100	100 × *k*_on_	blue
2	5 × *k*_on_	0.05	20	100 × *k*_on_	red
3	10 × *k*_on_	0.10	10	100 × *k*_on_	black

**Figure 2A:**
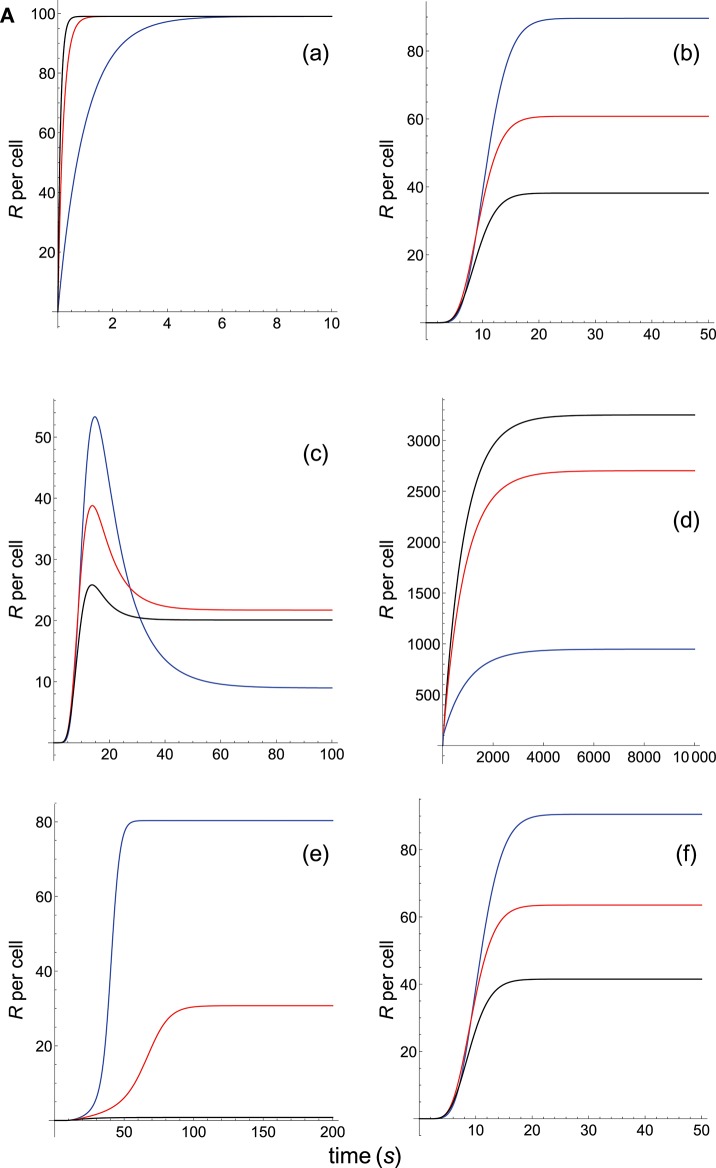
Dependence of the values of *R* on time for three systems with equal affinity (*A*) but different values of $k_{\text{on}}\left(s^{-1}\right)$ and $k_{\text{off}}\left(s^{-1}\right)$ given always in the form (*k*_on_,*k*_off_): system 1: (*k*_on_, 0.01), τ = 100*s*, *A* = 100*k*_on_ (blue); system 2: (5*k*_on_, 0.05), τ = 20*s*, *A* = 100*k*_on_ (red); system 3: (10*k*_on_, 0.10), τ = 10*s*, *A* = 100*k*_on_ (black). Panel models: **(a)** occupancy model; **(b)** basic kpr; **(c)** kpr with limited signaling; **(d)** kpr with sustained signaling; **(e)** kpr with negative feedback; **(f)** kpr with induced rebinding. Plots were obtained as described in main text and in Appendix. Number of pMHCs, *P*_*T*_ = 100. The values of the remaining parameters needed for computation in the different models are given in subsection 2.2.

**Figure 2B:**
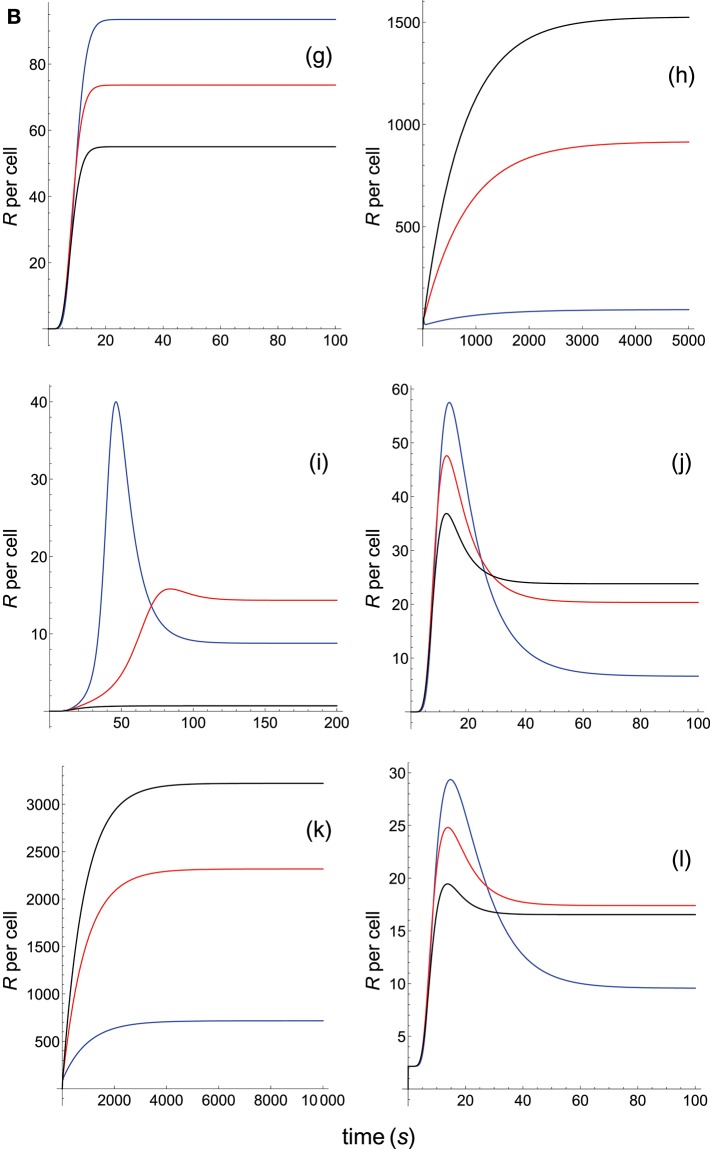
Dependence of the values of *R* on time for three systems with equal affinity (*A*) but different values of $k_{\text{on}}\left(s^{-1}\right)$ and $k_{\text{off}}\left(s^{-1}\right)$. Panel models: **(g)** kpr with stabilizing activation chain; **(h)** kpr with limited and sustained signaling; **(i)** kpr with negative feedback and limited signaling; **(j)** kpr with stabilizing activation chain and limited signaling; **(k)** kpr with stabilizing activation chain and sustained signaling; **(l)** kpr with limited signaling coupled to an incoherent feed-forward loop. Other conditions as in Figure 2A.

where the value of *k*_on_ is given in subsection 2.2.

The responses computed from the occupancy model are shown in panel a of Figure 2A. As expected, the transient phase is shorter as the *k*_on_-values increase, but at longer times, once the steady state is attained, the *R*-values become independent of the individual values of *k*_on_ and *k*_off_. Also, and because the three systems have equal affinity the corresponding steady-state responses show the same value in agreement with Equation (A1) in Appendix. However, in the basic kpr model (Figure 2A, panel **b**) the responses are quite different despite the three systems have the same affinity. Thus, the largest response is obtained for the system with the lowest values of *k*_on_ and *k*_off_ (blue curve). On the other hand, the responses for the kpr with limited signaling model (Figure 2A, panel **c**) are more complicated. As a result, the largest responses appear as peak values in transient phase with system 1 showing the largest value of *R*. However, as signaling progresses, the *R*-values decrease so that in steady state the response for system 1 becomes the lowest of the three systems. In turn, panel **d** of Figure 2A displays the *R*-values obtained for the kpr with sustained signaling model. In this case the largest responses for the three systems are attained in steady state, with system 3 (the system with the largest values of *k*_on_ and *k*_off_, black curve) producing the highest of the three *R*-values. Comparatively, we have that sustained signaling predicts much larger responses than those from others models^1^, although the time required to reach a steady state is also much longer. This behavior also occurs in those models that include this effect (panel **d** of Figure 2A, and panels **h** and **k** of Figure 2B). The response for the kpr with limited signaling coupled to an incoherent feed-forward loop model shows similar characteristics to those of the simpler kpr with limited signaling model (compare panel **l** of Figure 2B, and panel **c** of Figure 2A), although the *R*-values computed from the kpr-iff model are smaller. As discussed in Appendix, the maximum steady state response for the kpl-iff model is not limited by *T*_*T*_ or *P*_*T*_ but by *X*_*T*_. And although for comparative purposes we have considered *P*_*T*_ = *X*_*T*_ = 100, the lower responses of the kpl-iff model in comparison with those of the simpler kpl model are due to the presence of the iff loop and to the modulating effect of the activation chain *C*_0_→*C*_1_ → ⋯ → *C*_*N*_ on the signaling species *X*.

In any case, a clear pattern emerges from Figures 2A, B, namely: that, exception made of the occupancy model, systems with equal affinity show, however, quite different responses which would make affinity an unreliable estimate of the T-cell response. Further examples are given in Supplementary Material.

### 3.2. Systems With Different Affinities and Different Values of *k*_on_ and *k*_off_

In Figures 3A, B we have illustrated the responses computed from the 12 phenotypic models in three systems that have different affinities and the following values of *k*_on_ and *k*_off_:

**Table:** 

**System**	***k***_on_(s^−1^)	***k***_off_(s^−1^)	**τ (s)**	***A***	**Color curves in Figures 3A, B**
1	1000 × *k*_on_	1	1	1000 × *k*_on_	blue
2	10 × *k*_on_	0.1	10	100 × *k*_on_	red
3	0.1 × *k*_on_	0.01	100	10 × *k*_on_	black

**Figure 3A:**
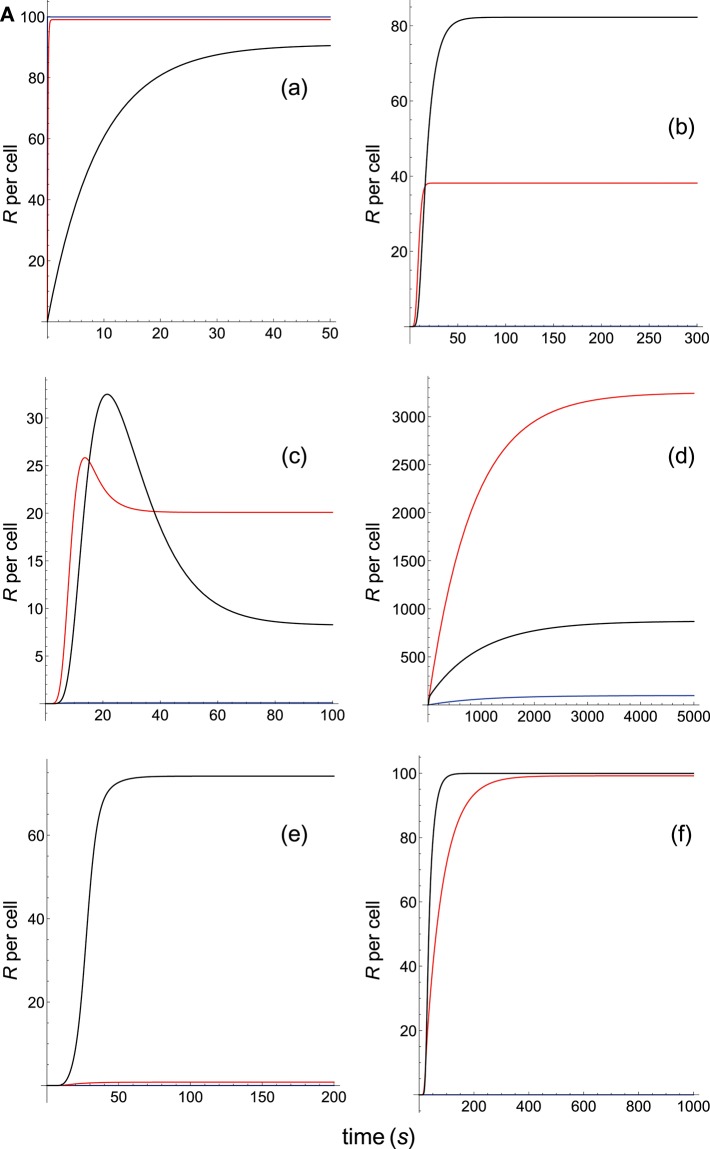
Dependence of the values of *R* on time for three systems with different affinities (*A*) and different values of $k_{\text{on}}\left(s^{-1}\right)$ and $k_{\text{off}}\left(s^{-1}\right)$ given in the form (*k*_on_,*k*_off_): system 1: (1000*k*_on_, 1), τ = 1*s*, *A* = 1000*k*_on_ (blue); system 2: (10*k*_on_, 0.10), τ = 10*s*, *A* = 100*k*_on_ (red); system 3: (0.1*k*_on_, 0.01), τ = 100*s*, *A* = 10*k*_on_ (black). Panel models: **(a)** occupancy model; **(b)** basic kpr; **(c)** kpr with limited signaling; **(d)** kpr with sustained signaling; **(e)** kpr with negative feedback; **(f)** kpr with induced rebinding. In some cases (v.g. **b**, blue curve), *R*-values are so small that their plots are almost coincident with the *x*-axis. Other conditions as in Figure 2A.

**Figure 3B:**
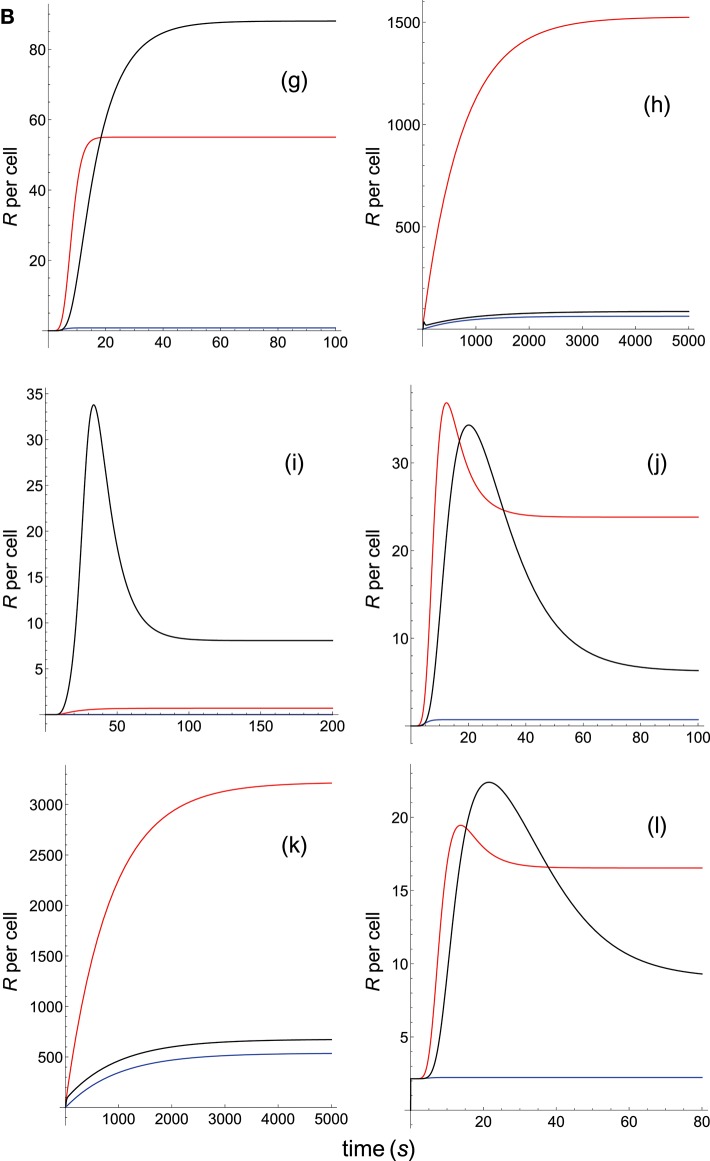
Dependence of the values of *R* on time for three systems with different affinities (*A*) and different values of $k_{\text{on}}\left(s^{-1}\right)$ and $k_{\text{off}}\left(s^{-1}\right)$. Panel models: **(g)** kpr with stabilizing activation chain; **(h)** kpr with limited and sustained signaling; **(i)** kpr with negative feedback and limited signaling; **(j)** kpr with stabilizing activation chain and limited signaling; **(k)** kpr with stabilizing activation chain and sustained signaling; **(l)** kpr with limited signaling coupled to an incoherent feed-forward loop. Other conditions as in Figures 2A, 3A.

where the value of *k*_on_ is given in subsection 2.2.

Panel **a** of Figure 3A shows that, as discussed in previous subsection and in Appendix, in the occupancy model responses increase always with affinity. Conversely, in the basic kpr model the system 3, with the smallest affinity, shows the largest response (panel **b** of Figure 3A, black curve), while in system 1, despite being the system with the largest affinity, practically no response is observed and its plot (blue curve) is almost coincident with the *x*-axis. Also, the following models: kpr with negative feedback (Figure 3A, panel **e**), kpr with induced rebinding (Figure 3A, panel **f**), kpr with stabilizing activation chain (Figure 3B, panel **g**), and kpr with negative feedback and limited signaling (Figure 3B, panel **i**), exhibit similar behavior to that observed for the basic kpr model, i.e., the largest response is obtained for the system with the lowest affinity (black curves) while null or very small values of *R* were found for the system with the largest affinity (blue curves). In turn, the kpr with limited signaling model displays a dual behavior (Figure 3A, Panel **c**): in transient phase the system with lowest affinity (black curve) shows the largest response, while in steady state the system with an intermediate value of *A* (red curve) gives the largest value of *R*. On the other hand, the system with the highest binding affinity practically shows no response at all times (blue curve). This behavior is also shown by the kpl-iff model (Figure 3B, panel **l**) although in this case the response for the system with the highest value of *A* coincides with the residual response in absence of kpr (see discussion in Appendix). In those models where sustained signaling is considered (panel **d** of Figure 3A and panels **h** and **k** of Figure 3B), we have that intermediate binding affinity gives the largest responses (red curves), the lowest affinity displays intermediate responses (black curves), while the system with the highest affinity (blue curves) shows the lowest *R*-values (although for the **h** and **k** models the *R*-values for the highest and lowest affinities are close, see Figure 3B).

Summarizing, we have that for the systems shown in this subsection only the occupancy model predicts a direct correlation between affinity and response independently of the values of *k*_on_ and *k*_off_. In the remaining models that correlation does not exist and thus, there are systems with low or intermediate binding affinity exhibiting larger responses than those with a higher affinity, or systems with low and high affinities giving very close responses.

### 3.3. Systems With Different Affinities but the Same Value of *k*_off_

In Figures 4A, B we have displayed the responses computed from the 12 phenotypic models in three systems that have equal *k*_off_ and increasing values of *k*_on_:

**Table:** 

**System**	***k***_on_(s^−1^)	***k***_off_(s^−1^)	**τ (s)**	***A***	**Color curves in Figures 4A, B**
1	0.01 × *k*_on_	0.1	10	0.1 × *k*_on_	Blue
2	*k*_on_	0.1	10	10 × *k*_on_	Red
3	100 × *k*_on_	0.1	10	1000 × *k*_on_	Black

**Figure 4A:**
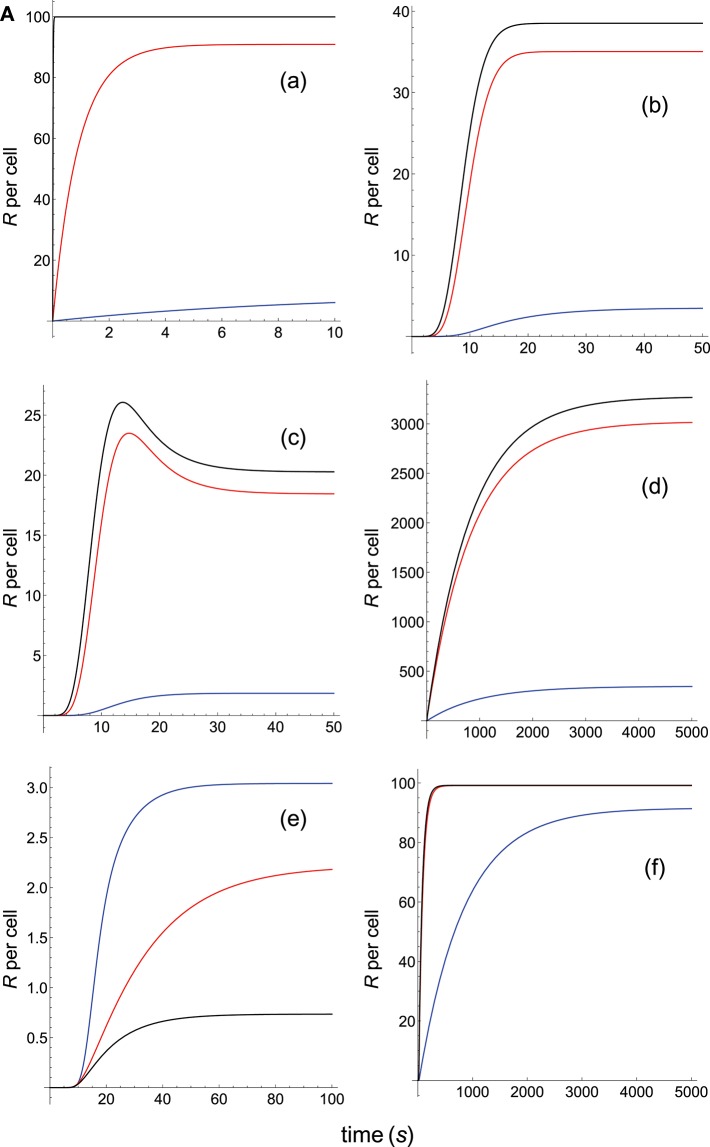
Dependence of the values of *R* on time for three systems with different affinities (*A*), different values of $k_{\text{on}}\left(s^{-1}\right)$ and equal values of $k_{\text{off}}\left(s^{-1}\right)$ given in the form (*k*_on_,*k*_off_): system 1: (0.01*k*_on_, 0.1), τ = 10*s*, *A* = 0.1*k*_on_ (blue); system 2: (*k*_on_, 0.1), τ = 10*s*, *A* = 10*k*_on_ (red); system 3: (100*k*_on_, 0.1), τ = 10*s*, *A* = 1000*k*_on_ (black). Panel models: **(a)** occupancy model; **(b)** basic kpr; **(c)** kpr with limited signaling; **(d)** kpr with sustained signaling; **(e)** kpr with negative feedback; **(f)** kpr with induced rebinding. Other conditions as in Figure 2A.

**Figure 4B:**
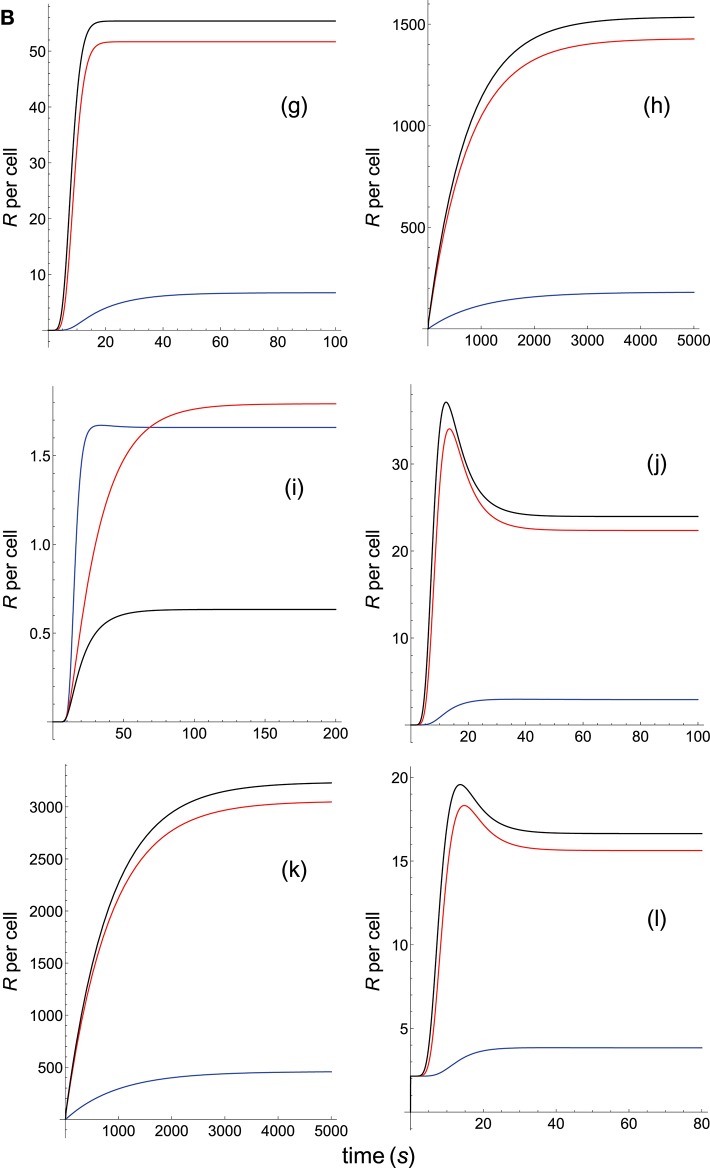
Dependence of the values of *R* on time for three systems with different affinities (*A*), different values of $k_{\text{on}}\left(s^{-1}\right)$ and equal values of $k_{\text{off}}\left(s^{-1}\right)$. Panel models: **(g)** kpr with stabilizing activation chain; **(h)** kpr with limited and sustained signaling; **(i)** kpr with negative feedback and limited signaling; **(j)** kpr with stabilizing activation chain and limited signaling; **(k)** kpr with stabilizing activation chain and sustained signaling; **(l)** kpr with limited signaling coupled to an incoherent feed-forward loop. Other conditions as in Figures 2A, 4A.

Because *k*_off_ is constant (τ = 10 s) the increase in affinity results only from the corresponding increase in the values of *k*_on_. Hence and, unlike the two previous sections where the computed values of *R* as a function of affinity were due to variations in both *k*_on_ and *k*_off_, the predicted responses in this section result only from the effect exerted by *k*_on_ (i.e., a single parameter), rather than by *A*. Therefore, since there are no trade-offs between *k*_on_ and *k*_off_ the data analysis of the corresponding responses is facilitated. Thus, in agreement with previous results, the occupancy model shows (Figure 4A, panel **a**) that under these conditions an increase in the values of *k*_on_ (and therefore, in affinity) produces an enhancement of the response. The same behavior is observed in the remaining panels of Figures 4A, B with the exception of the models in which negative feedback is involved (Figure 4A, panel **e**; Figure 4B, panel **i**), where systems with the largest values of *k*_on_ display the lowest responses (black curves). However, additional results for a larger value of τ (=100 s) given in Supplementary Material reveal that all models, including those with negative feedback, now show an increase of the response as *k*_on_ becomes larger. Further analysis of the results displayed in Figures 4A, B and in Supplementary Material also show that, whereas the remaining parameters are maintained constant, responses from all models become independent of the *k*_on_-values, and therefore of affinity, for *k*_on_>>1. In short, we have that all models (with the exception of the kpr with negative feedback model when τ = 10 s) predict that when *k*_off_ (or τ) is constant, the *R*-values increase with *k*_on_ and therefore, with affinity. But strictly speaking, what this data analysis really reveals is the effect exerted by variations in the values of the rate constant *k*_on_ on the predicted immune responses when the others parameters remain constant.

### 3.4. Systems With Different Affinities but the Same Value of *k*_on_

Immune responses exhibit a remarkable dependence on the values of *k*_off_(= 1/τ) (4, 37, 38, 56) and, in fact, it has been suggested that the value of *k*_off_ is the best estimate of T-cell activation (19, 57–59). To explore in further detail this dependence we have displayed in Figures 5A, B the responses computed from the 12 phenotypic models for five systems having equal *k*_on_ and decreasing values of *k*_off_ (increasing values of τ) for a relatively narrow interval of values of τ and *A*:

**Table:** 

**System**	***k***_on_(s^−1^)	***k***_off_(s^−1^)	**τ (s)**	***A***	**Color curves in Figures 5A, B**
1	*k*_on_	10	0.1	0.1 × *k*_on_	blue
2	*k*_on_	1	1	*k*_on_	red
3	*k*_on_	0.5	2	2 × *k*_on_	green
4	*k*_on_	0.2	5	5 × *k*_on_	magenta
5	*k*_on_	0.05	20	20 × *k*_on_	black

**Figure 5A:**
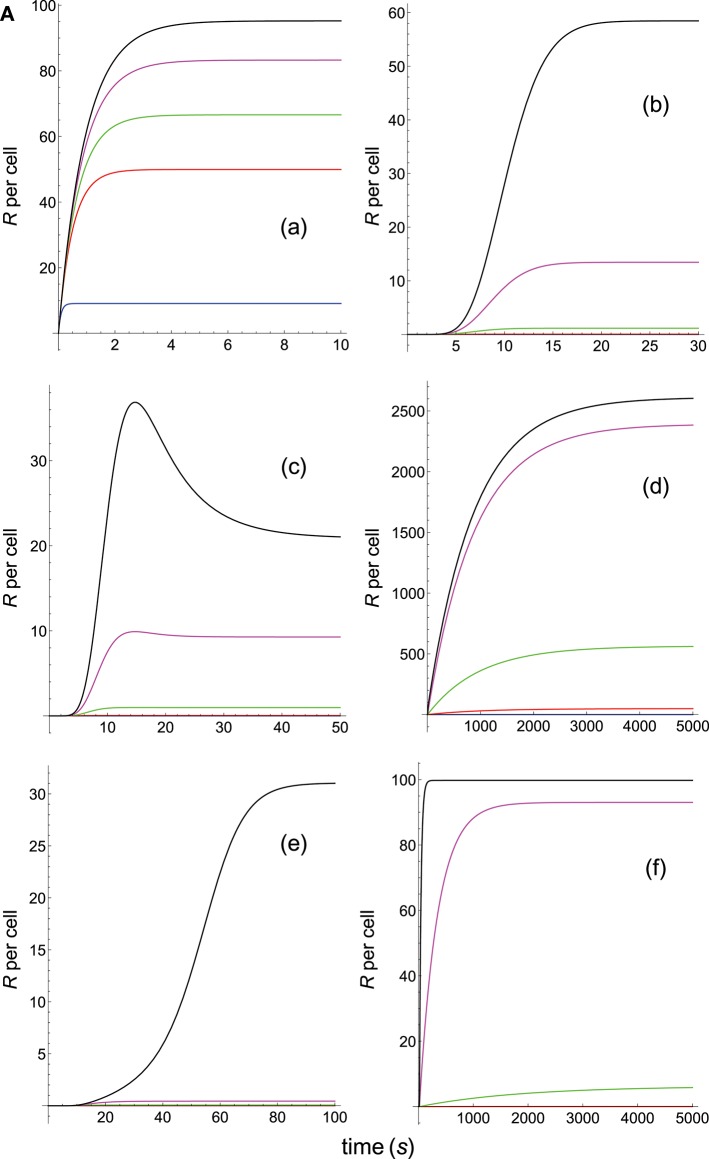
Dependence of the values of *R* on time for five systems with different affinities (*A*), different values of $k_{\text{off}}\left(s^{-1}\right)$ and equal values of $k_{\text{on}}\left(s^{-1}\right)$ given in the form (*k*_on_,*k*_off_): system 1: (*k*_on_, 10), τ = 0.1*s*, *A* = 0.1*k*_on_ (blue); system 2: (*k*_on_, 1), τ = 1*s*, *A* = *k*_on_ (red); system 3: (*k*_on_, 0.5), τ = 2*s*, *A* = 2*k*_on_ (green); system 4: (*k*_on_, 0.2), τ = 5*s*, *A* = 5*k*_on_ (magenta); system 5: (*k*_on_, 0.05), τ = 20*s*, *A* = 20*k*_on_ (black). Panel models: **(a)** occupancy model; **(b)** basic kpr; **(c)** kpr with limited signaling; **(d)** kpr with sustained signaling; **(e)** kpr with negative feedback; **(f)** kpr with induced rebinding. Other conditions as in Figure 2A.

**Figure 5B:**
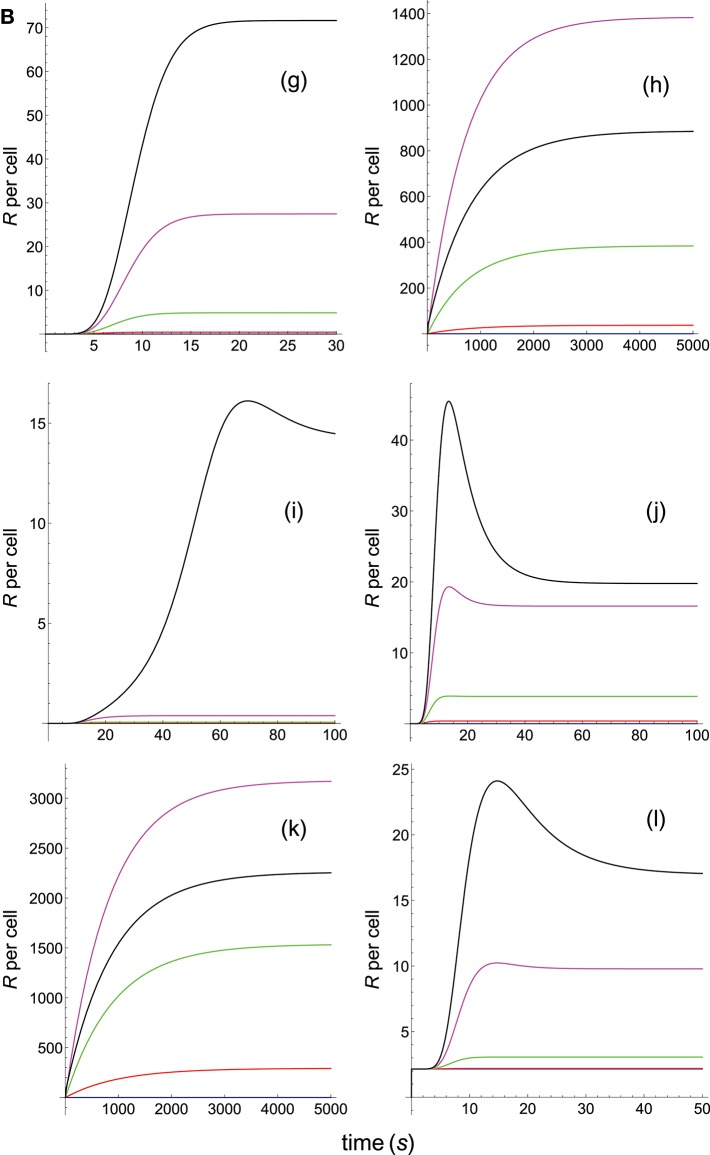
Dependence of the values of *R* on time for five systems with different affinities (*A*), different values of $k_{\text{off}}\left(s^{-1}\right)$ and equal values of $k_{\text{on}}\left(s^{-1}\right)$. Panel models: **(g)** kpr with stabilizing activation chain; **(h)** kpr with limited and sustained signaling; **(i)** kpr with negative feedback and limited signaling; **(j)** kpr with stabilizing activation chain and limited signaling; **(k)** kpr with stabilizing activation chain and sustained signaling; **(l)** kpr with limited signaling coupled to an incoherent feed-forward loop. Other conditions as in Figures 2A, 5A.

In turn, Figures 6A, B display the plots obtained for systems with a much wider range of τ and *A*-values:

**Table:** 

**System**	***k***_on_(s^−1^)	***k***_off_(s^−1^)	**τ (s)**	***A***	**Color curves in Figures 6A, B**
6	*k*_on_	0.02	50	50 × *k*_on_	blue
7	*k*_on_	0.01	100	100 × *k*_on_	red
8	*k*_on_	0.002	500	500 × *k*_on_	green
9	*k*_on_	0.001	1000	1000 × *k*_on_	magenta
10	*k*_on_	0.0001	10000	10000 × *k*_on_	black

**Figure 6A:**
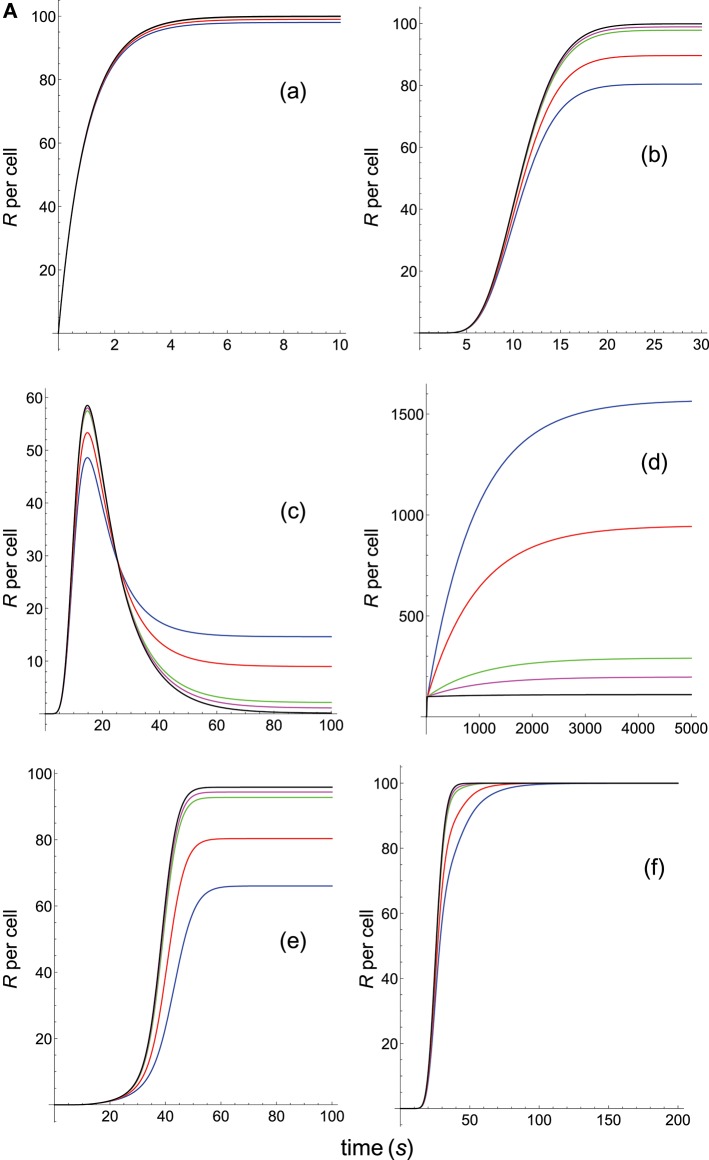
Dependence of the values of *R* on time for five systems with different affinities (*A*), different values of $k_{\text{off}}\left(s^{-1}\right)$ and equal values of $k_{\text{on}}\left(s^{-1}\right)$ given in the form (*k*_on_,*k*_off_): system 6: (*k*_on_, 0.02), τ = 50*s*, *A* = 50*k*_on_ (blue); system 7: (*k*_on_, 0.01), τ = 100*s*, *A* = 100*k*_on_ (red); system 8: (*k*_on_, 0.002), τ = 500*s*, *A* = 500*k*_on_ (green); system 9: (*k*_on_, 0.001), τ = 1000*s*, *A* = 1000*k*_on_ (magenta); system 10: (*k*_on_, 0.0001), τ = 10000*s*, *A* = 10000*k*_on_ (black). Panel models: **(a)** occupancy model; **(b)** basic kpr; **(c)** kpr with limited signaling; **(d)** kpr with sustained signaling; **(e)** kpr with negative feedback; **(f)** kpr with induced rebinding. Other conditions as in Figure 2A.

**Figure 6B:**
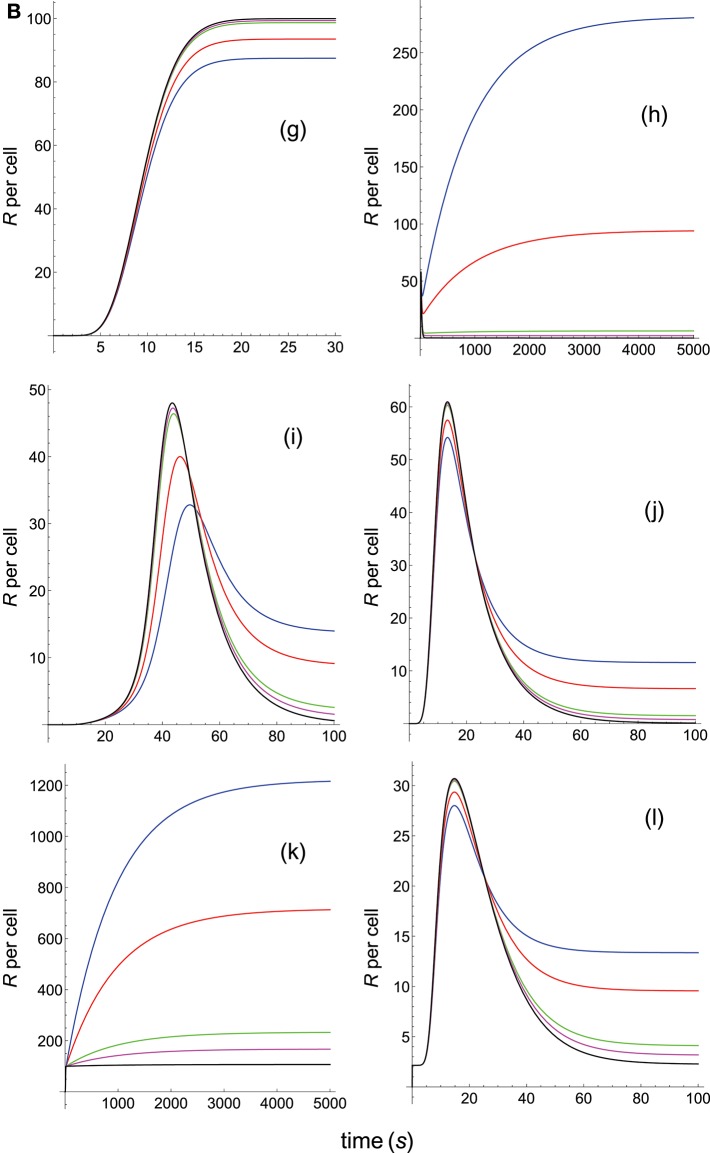
Dependence of the values of *R* on time for five systems with different affinities (*A*), different values of $k_{\text{off}}\left(s^{-1}\right)$ and equal values of $k_{\text{on}}\left(s^{-1}\right)$. Panel models: **(g)** kpr with stabilizing activation chain; **(h)** kpr with limited and sustained signaling; **(i)** kpr with negative feedback and limited signaling; **(j)** kpr with stabilizing activation chain and limited signaling; **(k)** kpr with stabilizing activation chain and sustained signaling; **(l)** kpr with limited signaling coupled to an incoherent feed-forward loop. Other conditions as in Figures 2A, 6A.

In Figures 5A–6B the value of *k*_on_ is constant so that increased affinity is only caused by the corresponding decreasing in the values of *k*_off_. Thus, we should bear in mind that plots in these Figures display in fact the effect exerted by a single parameter, *k*_off_ (or τ), rather than by *A*, on the predicted responses (in examining Figures 5A–6B, note that for some models and values of τ the corresponding *R*-values are so small that their plots are not observed because they almost coincide with the *x*-axis).

Panels **(a)** in Figures 5A, 6A display the *R*-values computed according to the occupancy model for the narrow and wide ranges of values of τ. Plots in these panels illustrate that, as already discussed for this model, values of *R* always increase as affinity becomes larger since they do not depend on the individual values of *k*_on_ and *k*_off_. The same behavior is observed for the basic kpr model [panels **(b)** in Figures 5A, 6A].

Note, however, that the behavior shown by the basic kpr model in Figures 5Ab, 6Ab, namely that an increased affinity as result of an increase in the τ-values when *k*_on_ is constant leads to larger responses, contrasts with the behavior described previously for the same model in subsection 3.2, where systems with larger affinities produced lower values of *R* (panel **b** in Figure 3A). This is due to the fact that affinity is not a parameter but a quotient of two parameters, so that variations in the values of *A* can occur in one of the following three ways: (a) changes in the values of *k*_on_ while *k*_off_ remains constant; (b) changes in the values of *k*_off_ while *k*_on_ remains constant; and (c) changes in the values of both *k*_on_ and *k*_off_. However, these three ways of changing affinity are not equivalent and produce different responses. This is shown in the following simple example demonstrating that the *R*-values are quite different when a 5-fold increased affinity is caused by a 5-fold increase in the value of *k*_on_ while *k*_off_ remains constant, or when the same increased affinity is obtained by dividing by five the value of *k*_off_ while *k*_on_ is constant (other conditions as in Figure 2A):

**Table:** 

**Model**	***k***_on_(s^−1^)	***k***_off_(s^−1^)	**τ (s)**	***A***	**R per cell**
	*k*_on_	0.1	10	10*k*_on_	35.03
Basic kpr	5*k*_on_	0.1	10	50*k*_on_	37.79
	*k*_on_	0.02	50	50*k*_on_	80.42

Data analysis of *R*-values computed from other models as a function of τ is more complex. Thus, for the kpr with limited signaling model the *R*-values increase with affinity for the smaller τ-values both in transient phase and in steady state (panel **c** in Figure 5A). However, for the largest values of τ (panel **c** in Figure 6A) the *R*-values increase with affinity reaching peak values in the transient phase, but as the time activation progresses the curves cross over so that in steady state the system with the largest affinity exhibits the lowest response^2^. The same behavior is also exhibited by the kpr with limited signaling coupled to an incoherent feed-forward loop model (panels **l** in Figures 5B, 6B). However, for the following three models, kpr with negative feedback, induced rebinding, and stabilizing activation chain (panels **e**, **f** in Figures 5A, 6A, and panel **g** in Figures 5B, 6B), the corresponding responses show a similar behavior to that observed for the simpler occupation and basic kpr models, i.e., that the *R*-values increase with affinity for all values of τ when *k*_on_ is constant. Regarding the kpr with sustained signaling model, the corresponding responses are more elaborate: the values of *R* increase with affinity for the smaller values of τ (panel **d** of Figure 5A), while conversely for the larger values of τ the opposite effect occurs (panel **d** of Figure 6A).

Finally, in the mixed models **(h)-(k)** the responses obtained are determined by the parameters and new assumptions added to the basic kpr model. For example, for the kpr with limited and sustained signaling model (where both effects modulate the response), the largest value of *R* in panel **h** of Figure 5B is not obtained for the system with the largest affinity (*A* = 20*k*_on_, black curve) but for the system with *A* = 5*k*_on_ (magenta curve). This contrast with the behavior observed under the same conditions for the kpr with only limited signaling model (Figure 5A, panel **c**), and the kpr with only sustained signaling model (Figure 5A, panel **d**) where the largest response in both cases was obtained for the largest affinity (black curves). These mixed characteristics are also observed quantitatively: the values of *R* in the mixed model (panel **h** in Figure 5B) are much larger than in Figure 5Ac (only limited signaling), but lower than in Figure 5Ad (only sustained signaling). Responses exhibiting combined behaviors are also observed for the other mixed models (panels **i, j, k**) in Figures 5B, 6B.

It is also worthy of note that comparison of responses from this and previous section shows that the influence exerted by the rate constant *k*_off_ is more involved than that exerted by *k*_on_. This is not surprising since Figures 1A, B show that the role of *k*_on_ in the reaction schemes of all models is limited to the first step of the activation chain, while conversely *k*_off_ is involved in most of the steps of the activation process. This is in agreement with experimental studies which had revealed the predominant influence exerted by τ on T-cell responses (19, 57–59).

Summarizing: results in this and previous sections allow to assess that the relationship between affinity and predicted T-cell responses is highly complex so that there is no a general and simple correlation between affinity (which is the quotient of two parameters) and response. Obviously, this does not exclude that for some systems with a particular set of parameters, or systems whose parameters are within a particular interval, a certain degree of correlation could be found.

### 3.5. Influence Exerted by the Concentration of Ligand

TCR-pMHC affinity (= *k*_on_/*k*_off_) is independent of the ligand concentration. However, it is expected that quantitative values of T-cell responses depend on the values of *P*_*T*_ and *T*_*T*_, the total amount of pMHC and TCR respectively. Therefore, the question arises as to whether data analysis from experimental studies aimed at establishing a correlation between affinity and T-cell response could be influenced by the values of *P*_*T*_ used in the assays. To this end, we have reproduced Figures from previous sections using a different *P*_*T*_-value ($P_{T}=2\times 10^{4}$ instead of *P*_*T*_ = 100), and the results are shown in Supplementary Material (SM). Thus, for systems with the same affinity but different values of *k*_on_ and *k*_off_ (Figures 2A, B in main text and the corresponding Figures 2AS, 2BS in Supplementary Material), in general we find that the behavior of the responses obtained from most models for both values of *P*_*T*_ were similar to those already discussed in subsection 3.1 of main text, although quantitative responses were much larger in the Figures displayed in SM. However, interesting exceptions were also found. For example, the kpr with sustained signaling model predicts that for the lower value of *P*_*T*_ responses for the three systems with the same affinity but different values of both rate constants are also different (panel **d** in Figure 2A in main text). However, the same model for $P_{T}=2\times 10^{4}$ shows that for the three systems with equal affinity the values of *R* are now practically equal (panel **d** in Figure 2AS in Supplementary Material). A superficial observation could state that “systems with equal affinity give the same response.” This, however, is a false conclusion because what this model really predicts is that under these conditions (a high value of *P*_*T*_) the three systems saturate and the maximum responses are attained independently of the values of affinity.

At the other end, we have that the kpr with negatived feedback model predicts extremely low or null responses for this high value of *P*_*T*_ (compare panels **e** of Figure 2A, Figure 2AS in main text and SM respectively). Again, this is not related to affinity but with the fact that for some values of the parameters (among them the *P*_*T*_-values) this mechanism can act as a switch so that for parameter-values below this switch the signaling chain goes forward (larger signaling) while above goes backward (no response).

In turn, inspection of the responses for the kpr with limited signaling coupled to an incoherent feed-forward loop model shows (compare panels **l** in Figure 2B, Figure 2BS): (a) that for *P*_*T*_ = 100 the values of *R* in steady state for the three systems increase in this order blue → black → red, while for $P_{T}=2\times 10^{4}$ we have red → black → blue, i.e., the *R*-values are reversed; (b) that unlike other models which predict much higher responses when $P_{T}=2\times 10^{4}$, the *R*-values in panel **l** of Figure 2B (*P*_*T*_ = 100, main text) and in panel **l** of Figure 2BS ($P_{T}=2\times 10^{4}$, SM) are always < 100. The reason, as shown in Appendix, is that responses for this mechanism are not bounded by *P*_*T*_ or *T*_*T*_ but by *X*_*T*_, the total amount of species *X*. Hence, and because in our modeling we have assumed *X*_*T*_ = 100, responses for this model can't be larger than this value independently of the values of *P*_*T*_, *T*_*T*_, or affinity.

Regarding the models built by adding assumptions and parameters from others models (models **h-k**) we notice that, effectively, responses can be modulated by these assumptions and parameters. Thus, for example, for the kpr with negative feedback and limited signaling model we find that for $P_{T}=2\times 10^{4}$ a null response is obtained (compare panels **i** of Figure 2B, Figure 2BS in main text and in SM) which reveals the strong effect exerted by the negative feedback process under these conditions (see above). Likewise, the kpr with stabilizing activation chain and sustained signaling model also shows the modulating effect exerted by the sustained signaling process (compare panels **k** of Figure 2B, Figure 2BS in main text and in SM).

Similar considerations apply also for the remaining Figures with $P_{T}=2\times 10^{4}$ displayed in SM, and for this reason further discussion is not given here.

### 3.6. T-cell Response and Parameter Space

In previous sections we have highlighted that to study without ambiguity the influence of a given parameter on the T-cell response we should analyze that response by varying only this parameter while all other parameters of the system are kept constant. Thus, if T-cell responses as a function of affinity are measured under conditions in which other system parameters also vary there will be cross-effects of these parameters on the observed response that mask the influence exerted by affinity. This could lead to misinterpret the results obtained and lead to false conclusions. In this section we illustrate a representative example of this situation by considering three different systems that have equal values of *k*_on_ and *k*_off_ and, therefore, of affinity, but they have different values of the rate constant along the proofreading activation chain, *k*_*p*_:

**Table:** 

**System**	***k***_on_(s^−1^)	***k***_off_(s^−1^)	***A***	***k***_***p***_(s^−1^)	**Color curves in Figures 7A, B**
1	*k*_on_	0.1	10 × *k*_on_	1	blue
2	*k*_on_	0.1	10 × *k*_on_	0.5	red
3	*k*_on_	0.1	10 × *k*_on_	0.1	black

**Figure 7A:**
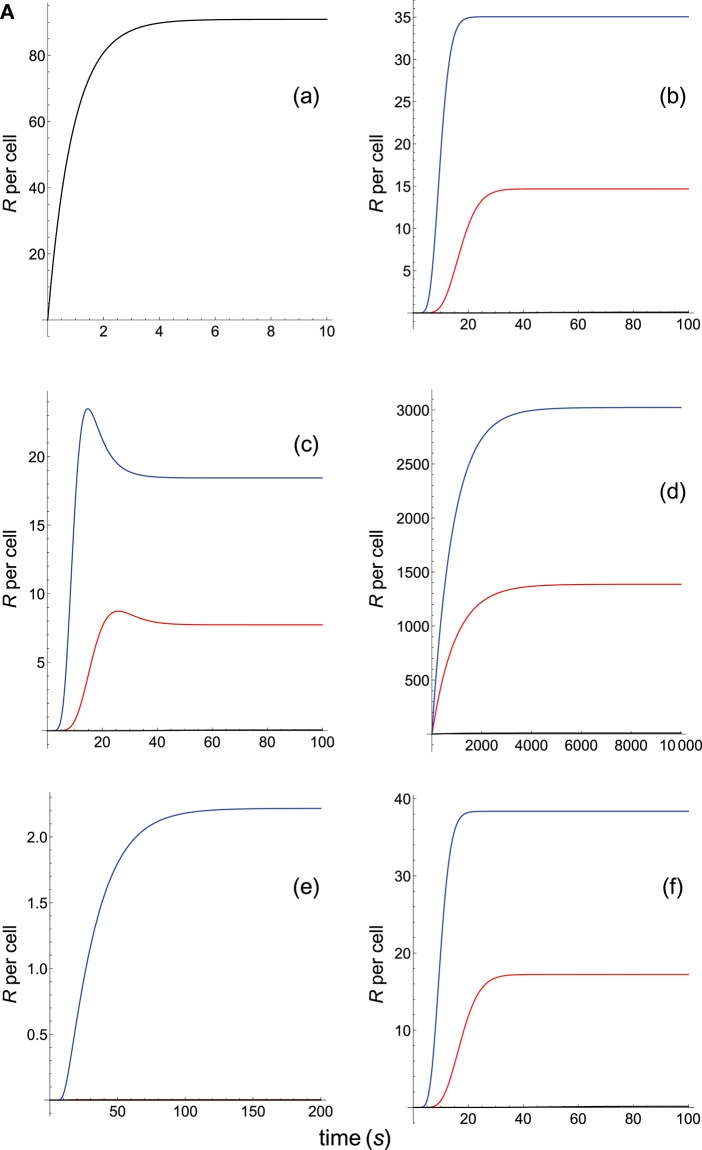
Dependence of the values of *R* on time for three systems with equal affinity (*A* = 10*k*_on_, $k_{\text{off}}=0.1s^{-1}$), but different values of $k_{p}\left(s^{-1}\right)$: system 1: *k*_*p*_ = 1 (blue); system 2: *k*_*p*_ = 0.5 (red); system 3: *k*_*p*_ = 0.1 (black). Panel models: **(a)** occupancy model; **(b)** basic kpr; **(c)** kpr with limited signaling; **(d)** kpr with sustained signaling; **(e)** kpr with negative feedback; **(f)** kpr with induced rebinding. In some cases, *R*-values are so small that their plots are almost coincident with the *x*-axis. Other conditions as in Figure 2A.

**Figure 7B:**
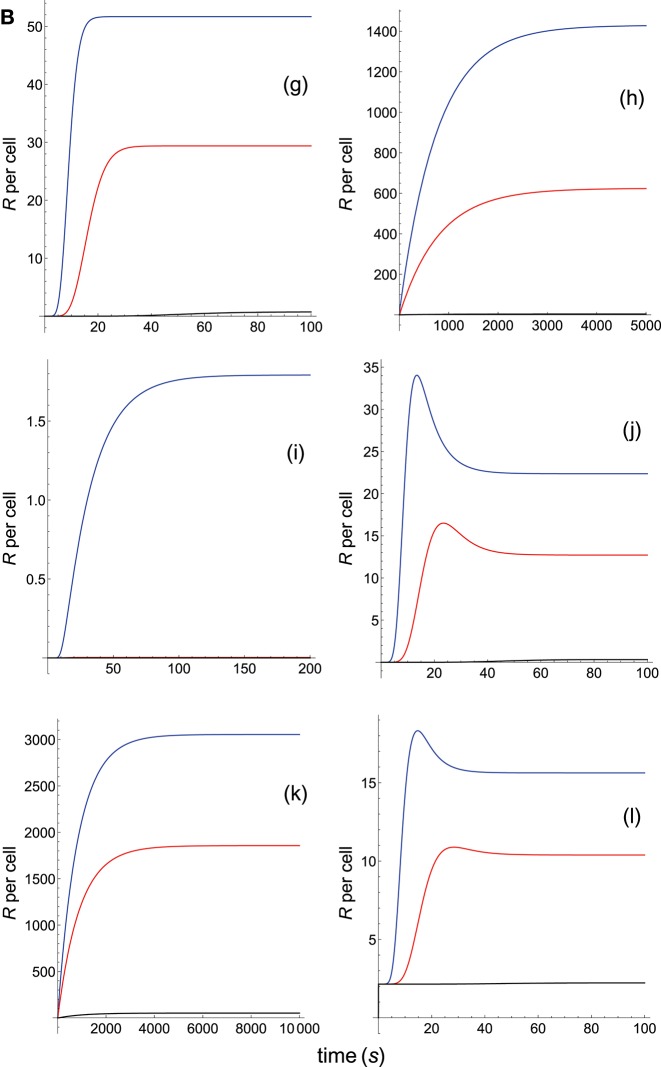
As in Figure 7A, but panel models are now: **(g)** kpr with stabilizing activation chain; **(h)** kpr with limited and sustained signaling; **(i)** kpr with negative feedback and limited signaling; **(j)** kpr with stabilizing activation chain and limited signaling; **(k)** kpr with stabilizing activation chain and sustained signaling; **(l)** kpr with limited signaling coupled to an incoherent feed-forward loop.

By assuming that we limit ourselves to consider T-cell responses and affinity, the responses from the three systems should be the same because they have equal affinity. For the occupancy model (Figure 7A, panel a) this is really the case because in this model only the binding process (which is independent of the rate constant *k*_*p*_) is involved. Hence, and since the values of *k*_on_, *k*_off_ and *A* are the same for the three systems, this model predicts that their responses are also equal and the three curves are overlaid. However, for the remaining models the predicted responses are quite different (Figures 7A, B, panels b-l) which, obviously, is due to the fact that computed responses were not obtained as previously in Figures 1–6, i.e., with all other system parameters remaining constant. This reveals that if T-cell measurements are analyzed without taken into account properly the parameter space it could lead to erroneous conclusions.

## 4. Conclusions

With exception of the extremely simple occupancy model, predicted T-cell responses from all models aimed at establishing the existence of a general positive correlation between affinity and T-cell response were negative which, in turn, could explain why a clear pattern of correlation between affinity and response has not been experimentally found (4, 7, 10, 12, 15, 17, 23). In fact, and leaving apart the occupancy model, it has been demonstrated in previous sections that none of the proposed models supports the existence of a correlation between affinity, the quotient of two parameters (*k*_on_/*k*_off_), and response. Rather, because both *k*_on_ and *k*_off_ are independently involved of each other in the response, we might consider the existence of correlations between the *R*-values and the individual rate constants *k*_on_ or *k*_off_, but not with their quotient. Hence, experiments designed to establish a correlation between affinity and immune response should be interpreted and analyzed with caution because, either these results are fortuitous or were obtained from systems with a particular set of parameters (or parameters within a particular interval) for which a certain degree of correlation could exist. These findings may be important in the design of adoptive T-cell immunotherapies based on producing high affinity TCR gene-modified T-cells against cancer antigens, or on attempting to determine the optimal receptor affinity for clinical effectiveness (20, 22, 29, 34, 35). Nevertheless, and in order to avoid confusion, experimental data analysis should clearly show that when a correlation is found is really due to the effect exerted by affinity, or it is rather the result of the effect exerted on the response by other parameters, v.g. *k*_on_ or τ.

But the above observations, and in particular those related to mixed models, raise also an interesting and more general issue: TCR-pMHC interaction is the keystone of the adaptive immune response, and this process exhibits an impressive capacity of speed, sensitivity and discrimination that allows to detect foreign pMHCs at very low concentration among much more abundant self-pMHC ligands (38, 60, 61). In addition, experimental studies concerning other characteristics of this process, namely the existence of an optimum dissociation time, dependence of this optimum τ on the pMHC concentration, correlation between pMHC potency (*EC*_50_) and the TCR-pMHC dissociation constant, and the relationship between maximum response and the binding parameters, among others, are far from conclusive and some are even contradictory (4). To this end, phenotypic models have been proposed to explain the above characteristics, but while some models are able to explain some of these facts they fail to explain others. Thus, and despite over three decades of intensive research, the mechanisms by which this remarkable interaction process determines the T-cell response remain controversial. But although this is not the subject of the present work, we would like to point out that assuming that phenotypic models can be built as modular systems, new models could be developed (such as some of the mixed models above described) to explain most of these characteristics. In this context, it has been recently suggested (62) that models for binding initiation of TCR signaling because they address or explain experimental observations are not necessarily mutually exclusive. Or, as shown in this work, that new phenotypic models can be built incorporating assumptions and parameters from others models in order to achieve better predictions and a better understanding of experimental observations.

## Author Contributions

JG and PG-P designed research, performed research, formulation of the model, analyzed data, and wrote the paper. JJG formulation of the model and analyzed data.

### Conflict of Interest Statement

The authors declare that the research was conducted in the absence of any commercial or financial relationships that could be construed as a potential conflict of interest.

## Footnotes

^1^This results from contribution of species *T*^*^, signaling competent TCRs after pMHC unbinding, to the response, see Figure 1A and Appendix.

^2^This shows that although most experimental studies on immune response are carried out in steady state conditions, if transient phase responses are measured data analysis could be different.
