# Supplementary material for: Is TCR/pMHC Affinity a Good Estimate of the T-cell Response? An Answer Based on Predictions From 12 Phenotypic Models
Source: Front Immunol. 2019 Mar 4;10:349. doi: 10.3389/fimmu.2019.00349 (PMC6410681; doi:10.3389/fimmu.2019.00349)
Supplement: Supplementary file 1 [file Presentation_1.pdf]

Is TCR/pMHC affinity a good estimate of the T-cell response? An answer based on predictions from twelve phenotypic models

Jesús Gálvez,[a,†], Juan J.Gálvez[b], and Pilar García-Peñarrubia[c]

[a]Department of Physical Chemistry, Faculty of Chemistry, University of Murcia, 30100 Murcia, Spain

[b]Department of Computer Science University of Illinois at Urbana-Champaign, 201 N Goodwin Ave, Urbana, IL 61801, EE.UU

[c]Department of Biochemistry and Molecular Biology B and Immunology, School of Medicine, University of Murcia, 30100 Murcia, Spain

[†]Corresponding author; e-mail: jgalvez@um.es

7. Appendix

7.1. Mathematical formulation of the models

In all models (see Figures 1A-1B) it is assumed that the response is related to the concentration of the corresponding productive signaling species[‡]. In model **(a)** this is the complex C_0, in models **(b)**, **(c)**, **(e)**, **(g)**, **(h)** and **(i)** the complex $C_N\,(N \geq 1)$, in models **(d)**, **(h)** and **(k)** the complex $C_N\,(N \geq 1)$ and T^*, in model **(f)** C_N and $C_N^*\,(N \geq 1)$, and in model **(l)** species X. The kinetic transient phases of these models are described by systems of ordinary differential equations (ODEs) some of which are shown here and the rest in Supplementary Material.

-Occupancy model (a):

In this simple model (panel **a** of Figure 1A) the predicted T-cell response is given by $R = C_0$ where C_0 is the concentration of the complex TCR-pMHC which is governed by the following differential equation

$$\frac{dC_0}{dt} = k_{\text{on}} PT - k_{\text{off}} C_0 \tag{A1}$$

where P and T are the concentrations of free pMHC and TCR respectively. Eqn.(A1) can be solved numerically using the initial condition

$$t = 0: \quad P = P_T\,,\, T = T_T\,,\, C_0 = 0 \tag{A2}$$

and the conservation equations

$$t > 0: \; P_T = P + C_0\;;\; T_T = T + C_0 \tag{A3}$$

with P_T and T_T being the total amount of pMHC and TCR. An analytical steady-state

[‡]for simplicity, we shall use the same notation to represent species and their concentrations

solution can be derived by inserting into eqn.(A1) the condition $dC_0/dt = 0$ and using eqns.(A3). Thus, we find

$$R = \frac{2P_T T_T}{P_T + T_T + 1/A + \sqrt{(P_T + T_T + 1/A)^2 - 4P_T T_T}} \tag{A4}$$

where $A = k_{\text{on}}/k_{\text{off}}$ is the binding affinity. As we will see below, this expression shows that the occupancy model is unique in the sense that for given values of P_T and T_T the values of R under steady state conditions are only a function of the TCR-pMHC affinity and not of the individual values of k_{on} and/or k_{off}, i.e. that systems with the same affinity should provide the same response. In addition, from eqn.(A4) we have

$$\lim_{A \to 0} R = 0 \;\; ; \;\; \lim_{A \to \infty} R = \begin{cases} T_T, \text{ if } P_T > T_T \\ P_T, \text{ if } T_T > P_T \end{cases} \tag{A5}$$

so that R-values increase always with A until a maximum value (bounded by T_T or P_T) is attained. Finally, eqn.(A4) shows that for a given value of A the upper bounds of R are also attained if assays are carried out with $P_T >> 1$ or $T_T >> 1$.

-kpr with stabilizing activation chain (g):

The kinetic transient phase kinetic of this model (panel **g** of Figure 1B) is described by the following system of ODEs:

$$\frac{dP}{dt} = -k_{\text{on}} PT + \sum_{i=0}^{N} k_{\text{off}}(i) C_i \tag{A6}$$

$$\frac{dT}{dt} = -k_{\text{on}} PT + \sum_{i=0}^{N} k_{\text{off}}(i) C_i \tag{A7}$$

$$\frac{dC_0}{dt} = k_{\text{on}} PT - (k_{\text{off}}(0) + k_p(0)) C_0 \tag{A8}$$

$$\frac{dC_i}{dt} = k_p(i-1)C_{i-1} - (k_{\text{off}}(i) + k_p(i))C_i \; ; \; 1 \le i \le N-1 \tag{A9}$$

$$\frac{dC_N}{dt} = k_{p(N-1)}C_{N-1} - k_{\text{off}(N)}C_N \tag{A10}$$

The parameters that govern the activation chain are N the number of steps leading to the productive signaling complex C_N, the binding rate k_{on}, and the dissociation and propagation rates $k_{\text{off}}(i)$ and $k_p(i)$. The complexes involved in the activation chain are C_i $(i = 0, 1, \cdots, N)$ where C_0 is the complex formed by the reversible binding of P and T which through a series of chemical modifications leads to C_N. In this model, and for simplicity, $k_{\text{off}}(0)$ and $k_p(0)$ are denoted as k_{off} $(= 1/\tau)$ and k_p where τ is the dissociation time of the complex C_0 and k_p the propagation rate of the step $C_0 \to C_1$. Once the functions $k_{\text{off}}(i)$ and $k_p(i)$ are provided, for example by using the expressions (37):

$$k_{\text{off}}(i) = \frac{(1+i)}{(1+ri)} \, k_{\text{off}} \, , \; r > 1 \;\; ; \;\; k_p(i) = k_p r^i \, , \; r > 1 \tag{A11}$$

the response $R = C_N$ as time progresses is obtained by solving numerically the system of eqns.(A6)-(A10) with the initial conditions:

$$t = 0: \quad P = P_T \, , \; T = T_T \, , \; C_i = 0 \; (i = 0, 1, \cdots, N) \tag{A12}$$

The steady-state solution is derived by inserting into the system of ODEs the conditions $dP/dt = 0$ (or $dT/dt = 0$) and $dC_i/dt = 0$, and takin into account the conservation equations

$$P_T = P + C_T \; ; \; T_T = T + C_T \; ; \; C_T = \sum_{i=0}^{N} C_i \tag{A13}$$

Thus, we have (37):

$$C_0 = \frac{C_T}{\mu} \;\; ; \;\; C_i = \gamma_i \, C_0 \, , \; 1 \le i \le N-1 \;\; ; \;\; C_N = \delta C_T \tag{A14}$$

with

$$\mu = 1 + \frac{k_{p(N-1)}}{k_{\mathrm{off}(N)}}\gamma_{(N-1)} + \sum_{i=1}^{N-1}\gamma_i \tag{A15}$$

$$\gamma_i = \alpha_1 \times \cdots \times \alpha_i = \prod_{j=1}^{i}\alpha_i \;\; ; \;\; \alpha_i = \frac{k_{p(i-1)}}{k_{p(i)} + k_{\mathrm{off}(i)}} \tag{A16}$$

$$\delta = \frac{1}{\mu}\frac{k_{p(N-1)}}{k_{\mathrm{off}(N)}}\gamma_{(N-1)} \tag{A17}$$

and where C_T is the number of bound receptors or ligands ($= \sum_{i=0}^{N} C_i$) which is given by

$$C_T = \frac{2P_T T_T}{P_T + T_T + \epsilon + \sqrt{(P_T + T_T + \epsilon)^2 - 4P_T T_T}} \;\; ; \;\; \epsilon = \frac{1}{\mu}\frac{k_{\mathrm{off}(0)} + k_{p(0)}}{k_{\mathrm{on}}} \tag{A18}$$

From these equations it is clear that, unlike eqn.(A4) in the occupancy model, the steady state response $R = \delta C_T$ cannot be expressed in simple terms of affinity.

-kpr with limited and sustained signaling (h):

For this model (panel **h** of Figure 1B) the system of ODEs is given by:

$$\frac{dP}{dt} = -k_{\mathrm{on}}PT - k_{\mathrm{on}}PT^* + k_{\mathrm{off}}\sum_{i=0}^{N+1} C_i \tag{A19}$$

$$\frac{dT}{dt} = -k_{\mathrm{on}}PT + k_{\mathrm{off}}\sum_{i=0}^{N-1} C_i + k_{\mathrm{off}}C_{N+1} + \lambda T^* \tag{A20}$$

$$\frac{dC_0}{dt} = k_{\mathrm{on}}PT - (k_{\mathrm{off}} + k_p)C_0 \tag{A21}$$

$$\frac{dC_i}{dt} = k_p C_{i-1} - (k_{\mathrm{off}} + k_p)C_i \; ; \; 1 \le i \le N-1 \tag{A22}$$

$$\frac{dC_N}{dt} = k_p C_{N-1} - (k_{\mathrm{off}} + \phi)C_N + k_{\mathrm{on}}PT^* \tag{A23}$$

$$\frac{dC_{N+1}}{dt} = \phi C_N - k_{\mathrm{off}}C_{N+1} \tag{A24}$$

$$\frac{dT^*}{dt} = k_{\text{off}} C_N - k_{\text{on}} P T^* - \lambda T^* \tag{A25}$$

and the initial conditions:

$$t = 0: \quad P = P_T \,,\; T = T_T \,,\; C_i = 0 \; (i = 0, 1, \cdots, N+1) \,,\; T^* = 0 \tag{A26}$$

Eqns.(A19)-(A25) are solved numerically and the response at $t > 0$ is given by $R = C_N + T^*$.

The steady-state response is derived by proceeding as in previous models and we find:

$$R = \frac{(k_{\text{on}}(P_T - C_T) + k_{\text{off}} + \lambda) k_{\text{off}} \alpha^N C_T}{(\phi + k_{\text{off}} \alpha^N) k_{\text{on}} (P_T - C_T) + \lambda(\phi + k_{\text{off}})} \tag{A27}$$

where

$$\alpha = \frac{k_p}{k_p + k_{\text{off}}} \,,\; C_T = \frac{2 P_T T_T}{P_T + T_T + 1/A + \sqrt{(P_T + T_T + 1/A)^2 - 4 P_T T_T}} \tag{A28}$$

and being $A = k_{\text{on}}/k_{\text{off}}$. Eqns.(A27)-(A28) must be solved simultaneously to obtain R. Hence, for given values of P_T, T_T, and N the steady-state response is a complex function not only of affinity (through C_T) but of the individual values of k_{on}, k_{off}, k_p, ϕ, and λ.

-kpr with negative feedback and limited signaling (i):

For this model (panel **i** of Figure 1B) we have:

$$\frac{dP}{dt} = -k_{\text{on}} P T + \sum_{i=0}^{N+1} k_{\text{off}} C_i \tag{A29}$$

$$\frac{dT}{dt} = -k_{\text{on}} P T + \sum_{i=0}^{N+1} k_{\text{off}} C_i \tag{A30}$$

$$\frac{dC_0}{dt} = k_{\text{on}} P T + (b + \gamma S) C_1 - (k_{\text{off}} + k_p) C_0 \tag{A31}$$

$$\frac{dC_i}{dt} = k_p C_{i-1} - (k_{\text{off}} + k_p + b + \gamma S) C_i + (b + \gamma S) C_{i+1} \;;\; 1 \le i \le N-1 \tag{A32}$$

$$\frac{dC_N}{dt} = k_p C_{N-1} - (k_{\text{off}} + b + \gamma S + \phi) C_N \tag{A33}$$

$$\frac{dC_{N+1}}{dt} = \phi C_N - k_{\text{off}} C_{N+1} \tag{A34}$$

$$\frac{dS}{dt} = \alpha C_1 (S_T - S) - \beta S \tag{A35}$$

with the initial conditions

$$t = 0: \quad P = P_T\,,\, T = T_T\,,\, C_i = 0\ (i = 0, 1, \cdots, N+1)\,,\, S = S_T \tag{A36}$$

The response is given by $R = C_N$

-kpr with stabilizing activation chain and limited signaling (j):

The kinetic transient phase kinetic of this model (panel **j** of Figure 1B) is described by the following system of ODEs:

$$\frac{dP}{dt} = -k_{\text{on}} PT + \sum_{i=0}^{N+1} k_{\text{off}}(i) C_i \tag{A37}$$

$$\frac{dT}{dt} = -k_{\text{on}} PT + \sum_{i=0}^{N+1} k_{\text{off}}(i) C_i \tag{A38}$$

$$\frac{dC_0}{dt} = k_{\text{on}} PT - (k_{\text{off}}(0) + k_p(0)) C_0 \tag{A39}$$

$$\frac{dC_i}{dt} = k_p(i-1) C_{i-1} - (k_{\text{off}}(i) + k_p(i)) C_i\,;\ 1 \le i \le N-1 \tag{A40}$$

$$\frac{dC_N}{dt} = k_p(N-1) C_{N-1} - (k_{\text{off}}(N) + \phi) C_N \tag{A41}$$

$$\frac{dC_{N+1}}{dt} = \phi C_N - k_{\text{off}}(N+1) C_{N+1} \tag{A42}$$

with the initial conditions:

$$t = 0: \quad P = P_T\,,\, T = T_T\,,\, C_i = 0\ (i = 0, 1, \cdots, N+1) \tag{A43}$$

Eqns.(A37)-(A42) are solved numerically once the functions $k_{\text{off}}(i)$ and $k_p(i)$ are provided (see model **(g)**) and the response at $t > 0$ is given by $R = C_N$.

The steady-solution can be derived proceeding as previously in model **(g)**, and we find that the same expressions remain valid (eqns.(A14)-A(18)) although the meanings of δ and μ in those expressions are now

$$\delta = \frac{1}{\mu} \frac{k_{p(N\,-\,1)}}{k_{\text{off}\,(N)} + \phi} \gamma_{(N-1)} \tag{A44}$$

$$\mu = 1 + \left(1 + \frac{\phi}{k_{\text{off}\,(N\,+\,1)}}\right) \frac{k_{p(N\,-\,1)}}{k_{\text{off}\,(N)} + \phi} \gamma_{(N-1)} + \sum_{i=1}^{N-1} \gamma_i \tag{A45}$$

The kpr with stabilizing activation chain and limited signaling model described by eqns. (A37)-(A45) has been built by incorporating the limited signaling process to model **(g)**. This shows the modular nature of phenotypic models and so, if the rate constant related to the limited signaling process $\phi = 0$ the equations derived in this section become those obtained for model **(g)**.

-kpr with stabilizing activation chain and sustained signaling (k):

For this model we have (panel **k** of Figure 1B):

$$\frac{dP}{dt} = -k_{\text{on}} PT + \sum_{i=0}^{N} k_{\text{off}}(i) C_i - k_{\text{on}} PT^* \tag{A46}$$

$$\frac{dT}{dt} = -k_{\text{on}} PT + \sum_{i=0}^{N-1} k_{\text{off}}(i) C_i + \lambda T^* \tag{A47}$$

$$\frac{dC_0}{dt} = k_{\text{on}} PT - (k_{\text{off}}(0) + k_p(0)) C_0 \tag{A48}$$

$$\frac{dC_i}{dt} = k_p(i-1) C_{i-1} - (k_{\text{off}}(i) + k_p(i)) C_i \; ; \; 1 \le i \le N-1 \tag{A49}$$

$$\frac{dC_N}{dt} = k_p(N-1) C_{N-1} - k_{\text{off}}(N) C_N + k_{\text{on}} PT^* \tag{A50}$$

$$\frac{dT^*}{dt} = k_{\text{off}} C_N - k_{\text{on}} P T^* - \lambda T^* \tag{A51}$$

with the initial conditions:

$$t = 0: \quad P = P_T \,,\, T = T_T \,,\, C_i = 0 \; (i = 0, 1, \cdots, N) \,,\, T^* = 0 \tag{A52}$$

The response is given by $R = C_N + T^*$.

-kpr with limited signaling coupled to an incoherent feed-forward loop (l):

In this case (panel **l** of Figure 1B) the system of ODEs is given by

$$\frac{dP}{dt} = -k_{\text{on}} P T + k_{\text{off}} \sum_{i=0}^{N+1} C_i \tag{A53}$$

$$\frac{dT}{dt} = -k_{\text{on}} P T + k_{\text{off}} \sum_{i=0}^{N+1} C_i \tag{A54}$$

$$\frac{dC_0}{dt} = k_{\text{on}} P T - (k_{\text{off}} + k_p) C_0 \tag{A55}$$

$$\frac{dC_i}{dt} = k_p C_{i-1} - (k_{\text{off}} + k_p) C_i \,;\; 1 \le i \le N-1 \tag{A56}$$

$$\frac{dC_N}{dt} = k_p C_{N-1} - (k_{\text{off}} + \phi) C_N \tag{A57}$$

$$\frac{dC_{N+1}}{dt} = \phi C_N - k_{\text{off}} C_{N+1} \tag{A58}$$

$$\frac{dY}{dt} = \gamma_+^y (Y_T - Y) - \gamma_-^y Y + \lambda_i C_N (Y_T - Y) \tag{A59}$$

$$\frac{dX}{dt} = \gamma_+^x (X_T - X) - \gamma_-^x X + \delta_i Y (X_T - X) - \mu_i C_N X \tag{A60}$$

and the initial conditions:

$$t = 0: \quad P = P_T \,,\, T = T_T \,,\, C_i = 0 \; (i = 0, 1, \cdots, N+1) \,,\, X = X_0 \,,\, Y = Y_0 \tag{A61}$$

Species X and Y are involved in the incoherent feed-forward loop and their initial values, X_0 and Y_0, depend on the conditions that exist in the incoherent feed-forward loop before the kpr process is triggered. For example, we may consider that both X and Y are wholly in their inactive forms so that $X_0 = Y_0 = 0$. Alternatively, we may assume that an equilibrium between X and Y and their inactive forms already exists independently of any other process, i.e. $X_0 = X_T/(1+\gamma_-^x/\gamma_+^x)$, $Y_0 = Y_T/(1+\gamma_-^y/\gamma_+^y)$. Finally, we may also consider that when the kpr process begins a steady-state had been already reached in the iff loop:

$$X_0 = \frac{(1+\delta_h/(1+\gamma_-^y/\gamma_+^y))X_T}{1+\gamma_-^x/\gamma_+^x+\delta_h/(1+\gamma_-^y/\gamma_+^y)} \ , \ \delta_h = \delta_i Y_T/\gamma_+^x \ , \ Y_0 = \frac{Y_T}{1+\gamma_-^y/\gamma_+^y} \tag{A62}$$

In any case, once the initial conditions are specified, eqns.(A53)-(A60) are solved numerically and the transient phase response is given by $R = X$.

The steady-state response at $t >> 1$ is obtained using the condition $dZ/dt = 0$ (where Z represents any of the species involved in eqns.(A53)-(A60)) and we find:

$$C_N = \left(\frac{k_{\rm off}}{k_{\rm off}+\phi}\right)\alpha^N C_T \tag{A63}$$

$$Y = \frac{(1+\lambda_i C_N/\gamma_+^y)Y_T}{1+\gamma_-^y/\gamma_+^y+\lambda_i C_N/\gamma_+^y} \ , \ X = \frac{(1+\delta_i Y/\gamma_+^x)X_T}{1+\gamma_-^x/\gamma_+^x+\delta_i Y/\gamma_+^x+\mu_i C_N/\gamma_+^x} \tag{A64}$$

where α and C_T are defined as previously by eqns.(A28).

From the above equations four remarks follow: a) the initial conditions defined by the values of X_0 and Y_0 affect the transient phase of the process but, however, the steady-state response is independent of X_0 and Y_0; b) unlike all the others models, for a given value of T_T the maximum steady-state response is not limited by T_T but by X_T and thus, if $X_T = 0$ we have $R = 0$ independently of the values of P_T; c) even in the absence of kpr (i.e. when P_T and/or T_T are zero so that $C_N = 0$), there is always a residual steady-state response given by X_0 in eqn.(A62) (or by eqns.(A64) with $C_N = 0$); and d) plots of X *vs.*

t could display two stationary states depending on the initial conditions X_0 and Y_0. Thus, if $X_0 = 0$ a first state stationary appears as X increases until the value given by eqn.(A62) is reached. Then, as the activation chain $C_0 \to C_1 \to \cdots \to C_N$ progresses the values of X increases again until a second steady state defined by eqns.(A63)-(A64) is reached.
