# Supplementary material for: Is TCR/pMHC Affinity a Good Estimate of the T-cell Response? An Answer Based on Predictions From 12 Phenotypic Models
Source: Front Immunol. 2019 Mar 4;10:349. doi: 10.3389/fimmu.2019.00349 (PMC6410681; doi:10.3389/fimmu.2019.00349)
Supplement: Supplementary file 2 [file Data_Sheet_1.PDF]

Supplementary Material

Is TCR/pMHC affinity a good estimate of the T-cell response? An answer based on predictions from twelve phenotypic models

Jesús Gálvez,[a,†], Juan J.Gálvez[b], and Pilar García-Peñarrubia[c]

[a]Department of Physical Chemistry, Faculty of Chemistry, University of Murcia, 30100 Murcia, Spain

[b]Department of Computer Science University of Illinois at Urbana-Champaign, 201 N Goodwin Ave, Urbana, IL 61801, EE.UU

[c]Department of Biochemistry and Molecular Biology B and Immunology, School of Medicine, University of Murcia, 30100 Murcia, Spain

[†]Corresponding author; e-mail: jgalvez@um.es

1. Mathematical formulation of the models

The systems of ordinary differential equations (ODEs) that describe the kinetic transient phases of the phenotypic models not shown in Appendix (see Figure 1A-1B in main text) are:

-kinetic proof reading (kpr) (b):

$$\frac{dP}{dt} = -k_{\text{on}}PT + \sum_{i=0}^{N} k_{\text{off}}C_i \tag{1}$$

$$\frac{dT}{dt} = -k_{\text{on}}PT + \sum_{i=0}^{N} k_{\text{off}}C_i \tag{2}$$

$$\frac{dC_0}{dt} = k_{\text{on}}PT - (k_{\text{off}} + k_p)C_0 \tag{3}$$

$$\frac{dC_i}{dt} = k_pC_{i-1} - (k_{\text{off}} + k_p)C_i \;;\; 1 \leq i \leq N-1 \tag{4}$$

$$\frac{dC_N}{dt} = k_pC_{N-1} - k_{\text{off}}C_N \tag{5}$$

The response $R = C_N$ is obtained by solving numerically the system of eqns.(1)-(5) with the initial conditions:

$$t = 0 : \quad P = P_T \, , \, T = T_T \, , \, C_i = 0 \; (i = 0, 1, \cdots, N) \tag{6}$$

-kpr with limited signalling (c):

$$\frac{dP}{dt} = -k_{\text{on}}PT + k_{\text{off}} \sum_{i=0}^{N+1} C_i \tag{7}$$

$$\frac{dT}{dt} = -k_{\text{on}}PT + k_{\text{off}} \sum_{i=0}^{N+1} C_i \tag{8}$$

$$\frac{dC_0}{dt} = k_{\text{on}}PT - (k_{\text{off}} + k_p)C_0 \tag{9}$$

$$\frac{dC_i}{dt} = k_p C_{i-1} - (k_{\rm off} + k_p) C_i \; ; \; 1 \le i \le N-1 \tag{10}$$

$$\frac{dC_N}{dt} = k_p C_{N-1} - (k_{\rm off} + \phi) C_N \tag{11}$$

$$\frac{dC_{N+1}}{dt} = \phi C_N - k_{\rm off} C_{N+1} \tag{12}$$

and the initial conditions:

$$t = 0 : \quad P = P_T \, , \, T = T_T \, , \, C_i = 0 \; (i = 0, 1, \cdots, N+1) \tag{13}$$

The response is given by $R = C_N$.

-kpr with sustained signalling (d):

$$\frac{dP}{dt} = -k_{\rm on} PT + k_{\rm off} \sum_{i=0}^{N} C_i - k_{\rm on} PT^* \tag{14}$$

$$\frac{dT}{dt} = -k_{\rm on} PT + k_{\rm off} \sum_{i=0}^{N-1} C_i + \lambda T^* \tag{15}$$

$$\frac{dC_0}{dt} = k_{\rm on} PT - (k_{\rm off} + k_p) C_0 \tag{16}$$

$$\frac{dC_i}{dt} = k_p C_{i-1} - (k_{\rm off} + k_p) C_i \; ; \; 1 \le i \le N-1 \tag{17}$$

$$\frac{dC_N}{dt} = k_p C_{N-1} - k_{\rm off} C_N + k_{\rm on} PT^* \tag{18}$$

$$\frac{dT^*}{dt} = k_{\rm off} C_N - k_{\rm on} PT^* - \lambda T^* \tag{19}$$

and the initial conditions:

$$t = 0 : \quad P = P_T \, , \, T = T_T \, , \, C_i = 0 \; (i = 0, 1, \cdots, N) \, , \, T^* = 0 \tag{20}$$

The response is given by $R = C_N + T^*$.

-kpr with negative feedback (e):

$$\frac{dP}{dt} = -k_{\text{on}}PT + \sum_{i=0}^{N} k_{\text{off}}C_i \tag{21}$$

$$\frac{dT}{dt} = -k_{\text{on}}PT + \sum_{i=0}^{N} k_{\text{off}}C_i \tag{22}$$

$$\frac{dC_0}{dt} = k_{\text{on}}PT + (b + \gamma S)C_1 - (k_{\text{off}} + k_p)C_0 \tag{23}$$

$$\frac{dC_i}{dt} = k_pC_{i-1} - (k_{\text{off}} + k_p + b + \gamma S)C_i + (b + \gamma S)C_{i+1}\ ;\ 1 \leq i \leq N-1 \tag{24}$$

$$\frac{dC_N}{dt} = k_pC_{N-1} - (k_{\text{off}} + b + \gamma S)C_N \tag{25}$$

$$\frac{dS}{dt} = \alpha C_1(S_T - S) - \beta S \tag{26}$$

and the initial conditions

$$t = 0: \quad P = P_T\,,\ T = T_T\,,\ C_i = 0\ (i = 0, 1, \cdots, N)\,,\ S = S_T \tag{27}$$

The response is given by $R = C_N$

-kpr with induced rebinding (f):

The system of ODE for this model is (1)

$$\frac{dP}{dt} = -k_{\text{on}}PT + k_{\text{off}}C_0 + \lambda_r \sum_{i=0}^{N} C_i^* \tag{28}$$

$$\frac{dT}{dt} = -k_{\text{on}}PT + k_{\text{off}}C_0 + \lambda_r \sum_{i=0}^{N} C_i^* \tag{29}$$

$$\frac{dC_0}{dt} = k_{\text{on}}PT - (k_{\text{off}} + k_p)C_0 \tag{30}$$

$$\frac{dC_i}{dt} = k_pC_{i-1} - (k_{\text{off}} + k_p)C_i + \rho_i C_i^*\ ;\ 1 \leq i \leq N-1 \tag{31}$$

$$\frac{dC_N}{dt} = k_p C_{N-1} - k_{\rm off} C_N + \rho_n C_N^* \tag{32}$$

$$\frac{dC_1^*}{dt} = k_{\rm off} C_1 - (\rho_1 + \lambda_r + k_p) C_1^* \tag{33}$$

$$\frac{dC_i^*}{dt} = k_p C_{i-1}^* + k_{\rm off} C_i - (\rho_i + \lambda_r + k_p) C_i^* \; ; \; 2 \leq i \leq N-1 \tag{34}$$

$$\frac{dC_N^*}{dt} = k_p C_{N-1}^* + k_{\rm off} C_N - (\rho_N + \lambda_r) C_N^* \tag{35}$$

with the initial conditions:

$$t = 0: \quad P = P_T \, , \, T = T_T \, , \, C_i = 0 \, , \, C_i^* = 0 \; (i = 0, 1, \cdots, N) \tag{36}$$

The response is given by $R = C_N + C_N^*$.

2. Systems with equal affinity but different values of $k_{\rm on}$ and $k_{\rm off}$

The behavior of the response of systems with equal affinity but different values of $k_{\rm on}$ and $k_{\rm off}$ was discussed in subsection 3.1 of main text (Figures 2A-2B). Further examples are displayed in Supplementary Material as Figures 2AS-2BS.

3. Systems with different affinities but the same value of $k_{\rm off}$

Figures 4AS and 4BS in Supplementary Material have been obtained as Figures 4A and 4B in subsection 3.3 of main text but with $\tau = 100\,$s instead of $\tau = 10\,$s.

4. Influence exerted by the concentration of ligand

Figures 2A-6A and 2B-6B in main text were obtained with $P_T = 100$. The corresponding Figures 2AS(+)-6AS(+) and 2BS(+)-6BS(+) in Supplementary Material have been computed as Figures in main tex but with $P_T = 2 \times 10^4$ instead of $P_T = 100$ (all Figures with $P_T = 2 \times 10^4$ are marked with (+)).

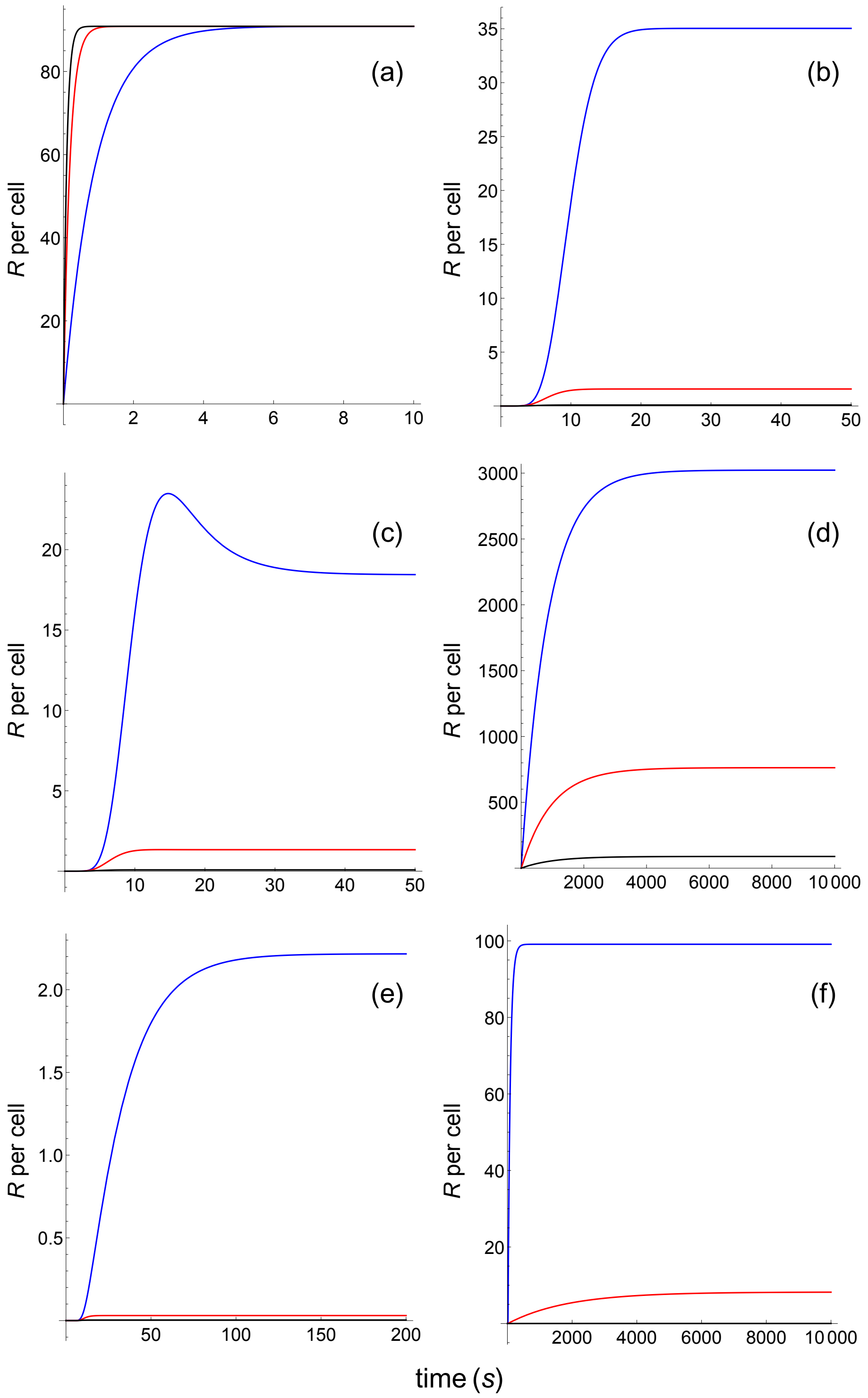

Figure 2AS: Dependence of the values of R on time for three systems with equal affinity (A) but different values of $k_{\rm on}\,({\rm s}^{-1})$ and $k_{\rm off}\,({\rm s}^{-1})$ given always in the form ($k_{\rm on}$,$k_{\rm off}$): system 1: ($k_{\rm on}$, 0.1), $\tau = 10\,{\rm s}$, $A = 10k_{\rm on}$ (blue); system 2: ($5k_{\rm on}$, 0.5), $\tau = 2\,{\rm s}$, $A = 10k_{\rm on}$ (red); system 3: ($10k_{\rm on}$, 1), $\tau = 1\,{\rm s}$, $A = 10k_{\rm on}$ (black). Panel models: **(a)** occupancy model; **(b)** basic kpr; **(c)** kpr with limited signalling; **(d)** kpr with sustained signalling; **(e)** kpr with negative feedback; **(f)** kpr with induced rebinding. Plots were obtained as described in main text and in Appendix. Number of pMHCs, $P_T = 100$. All remaining parameters values needed for computation in the different models are given in subsection 2.2 of main text.

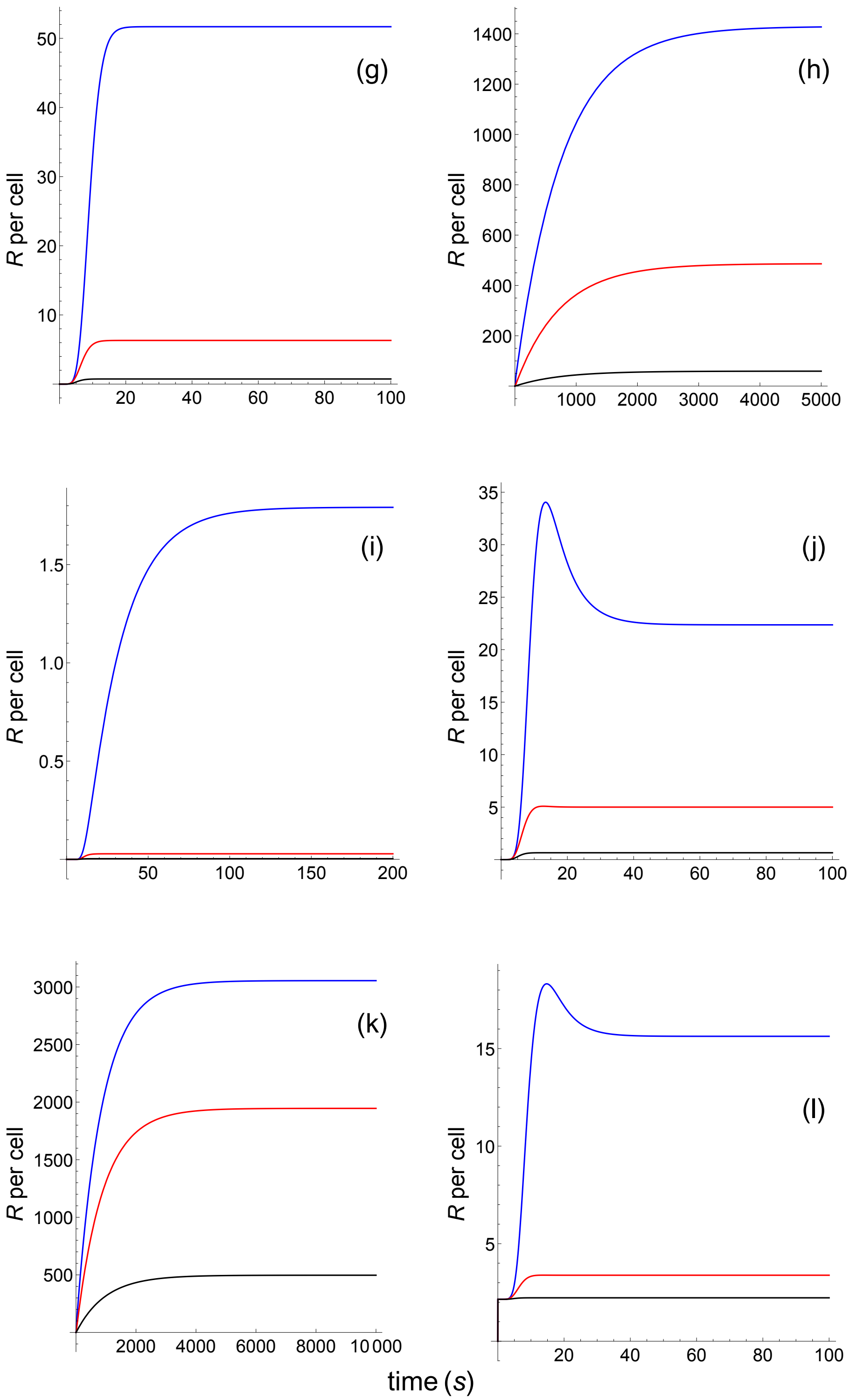

Figure 2BS: Dependence of the values of R on time for three systems with equal affinity (A) but different values of $k_{\mathrm{on}}\,(\mathrm{s}^{-1})$ and $k_{\mathrm{off}}\,(\mathrm{s}^{-1})$. Panel models: **(g)** kpr with stabilizing activation chain; **(h)** kpr with limited and sustained signalling; **(i)** kpr with negative feedback and limited signalling; **(j)** kpr with stabilizing activation chain and limited signalling; **(k)** kpr with stabilizing activation chain and sustained signalling; **(l)** kpr with limited signalling coupled to an incoherent feed-forward loop. Other conditions as in Figure 2AS.

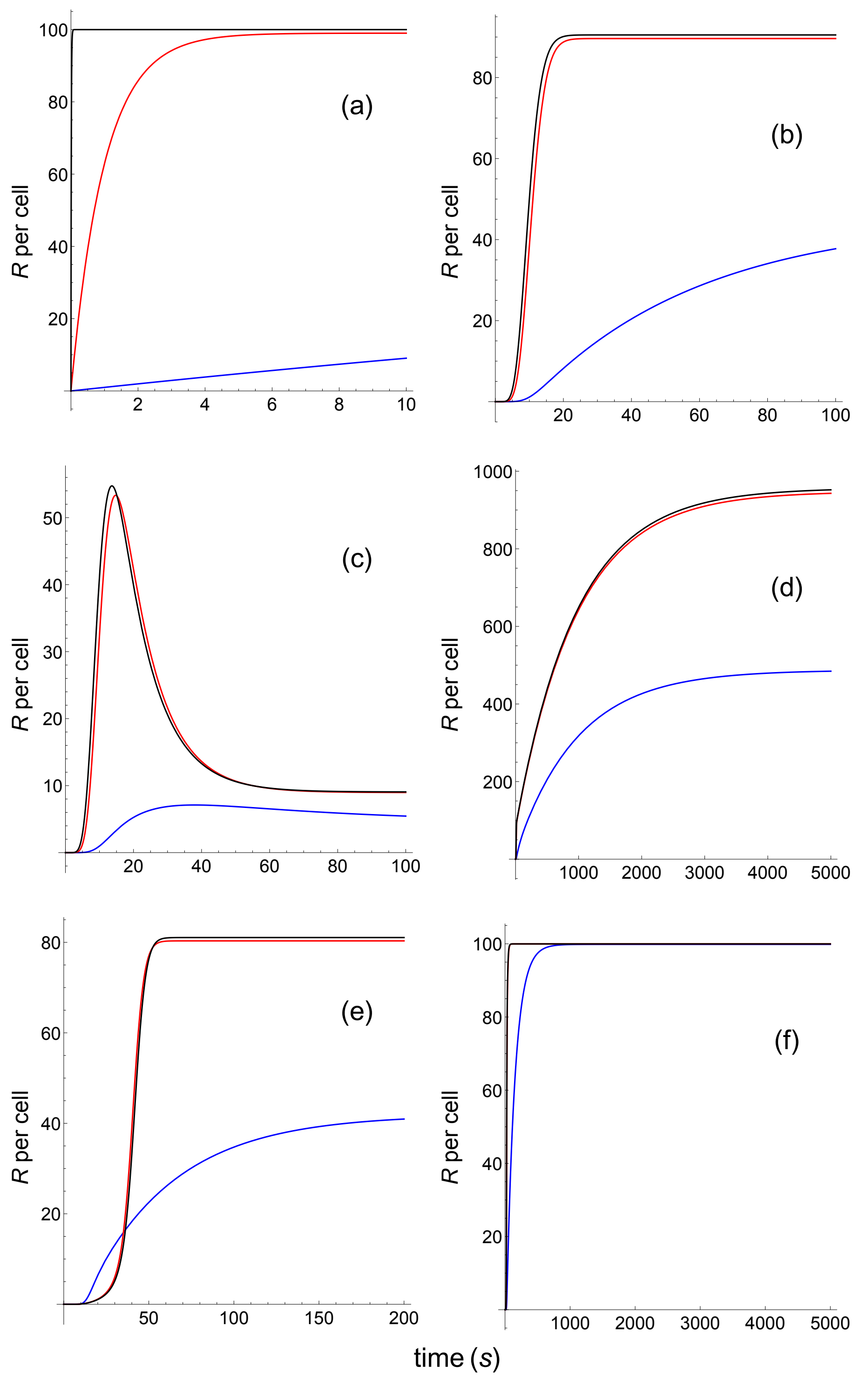

Figure 4AS: Dependence of the values of R on time for three systems with different affinities (A), different values of k_{on} (s^{-1}) and equal values of k_{off} (s^{-1}) given in the form (k_{on},k_{off}): system 1: ($0.01k_{\mathrm{on}}$, 0.01), $\tau = 100\,\mathrm{s}$, $A = k_{\mathrm{on}}$ (blue); system 2: (k_{on}, 0.01), $\tau = 100\,\mathrm{s}$, $A = 100k_{\mathrm{on}}$ (red); system 3: ($100k_{\mathrm{on}}$, 0.01), $\tau = 100\,\mathrm{s}$, $A = 10000k_{\mathrm{on}}$ (black). Panel models: **(a)** occupancy model; **(b)** basic kpr; **(c)** kpr with limited signalling; **(d)** kpr with sustained signalling; **(e)** kpr with negative feedback; **(f)** kpr with induced rebinding. Other conditions as in Figure 2AS.

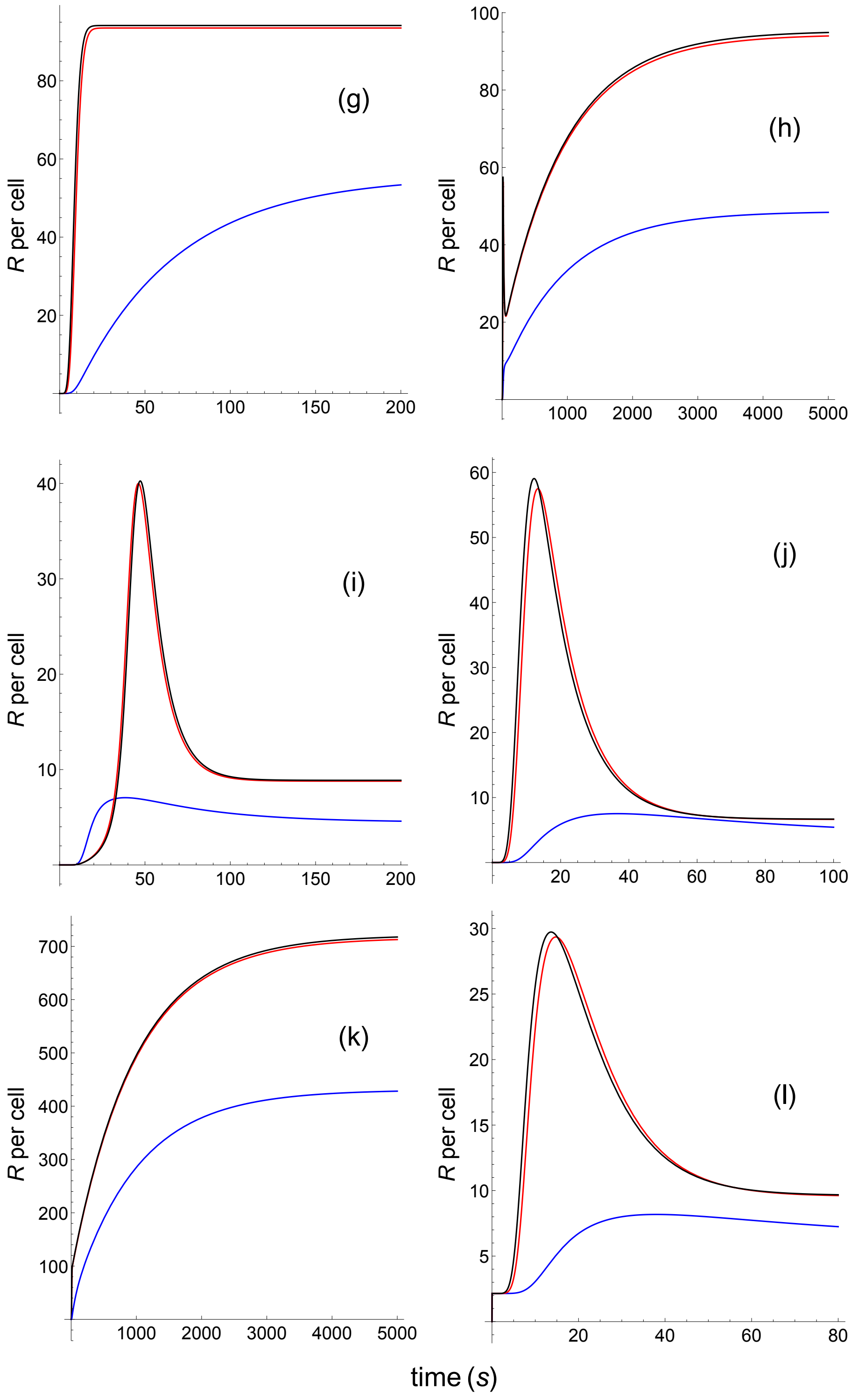

Figure 4BS: Dependence of the values of R on time for three systems with different affinities (A), different values of $k_{\mathrm{on}}\ (\mathrm{s}^{-1})$ and equal values of $k_{\mathrm{off}}\ (\mathrm{s}^{-1})$. Panel models: **(g)** kpr with stabilizing activation chain; **(h)** kpr with limited and sustained signalling; **(i)** kpr with negative feedback and limited signalling; **(j)** kpr with stabilizing activation chain and limited signalling; **(k)** kpr with stabilizing activation chain and sustained signalling; **(l)** kpr with limited signalling coupled to an incoherent feed-forward loop. Other conditions as in Figures 4AS and 2AS.

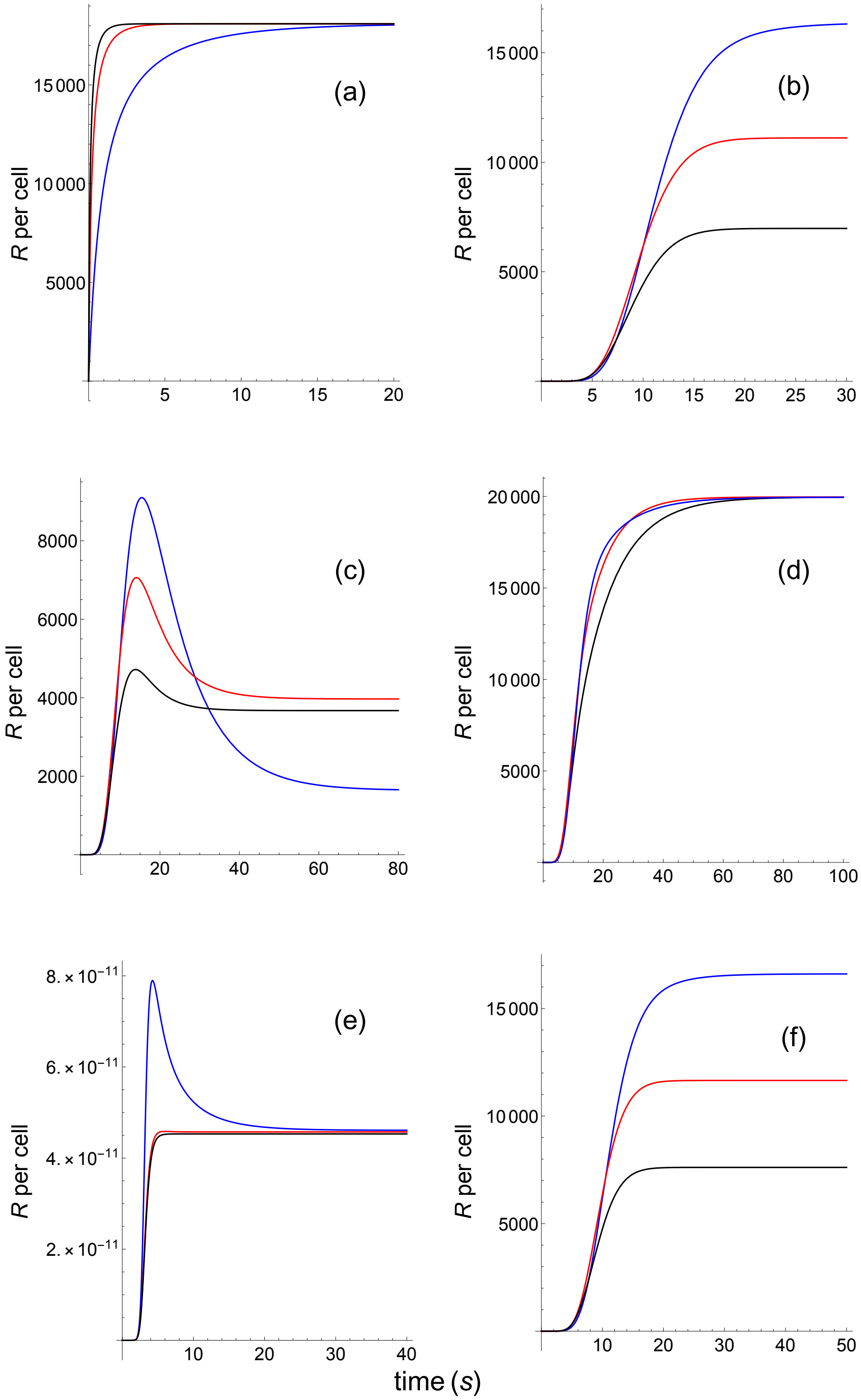

Figure 2AS(+): Dependence of the values of R on time for three systems with equal affinity (A) but different values of $k_{\text{on}}\,(\text{s}^{-1})$ and $k_{\text{off}}\,(\text{s}^{-1})$ given always in the form (k_{on},k_{off}): system 1: (k_{on}, 0.01), $\tau = 100\,\text{s}$, $A = 100k_{\text{on}}$ (blue); system 2: ($5k_{\text{on}}$, 0.05), $\tau = 20\,\text{s}$, $A = 100k_{\text{on}}$ (red); system 3: ($10k_{\text{on}}$, 0.10), $\tau = 10\,\text{s}$, $A = 100k_{\text{on}}$ (black). Panel models: **(a)** occupancy model; **(b)** basic kpr; **(c)** kpr with limited signalling; **(d)** kpr with sustained signalling; **(e)** kpr with negative feedback; **(f)** kpr with induced rebinding. Plots were obtained as described in main text and in Appendix. Number of pMHCs, $P_T = 2 \times 10^4$. All remaining parameters values needed for computation in the different models are given in subsection 2.2 of main text.

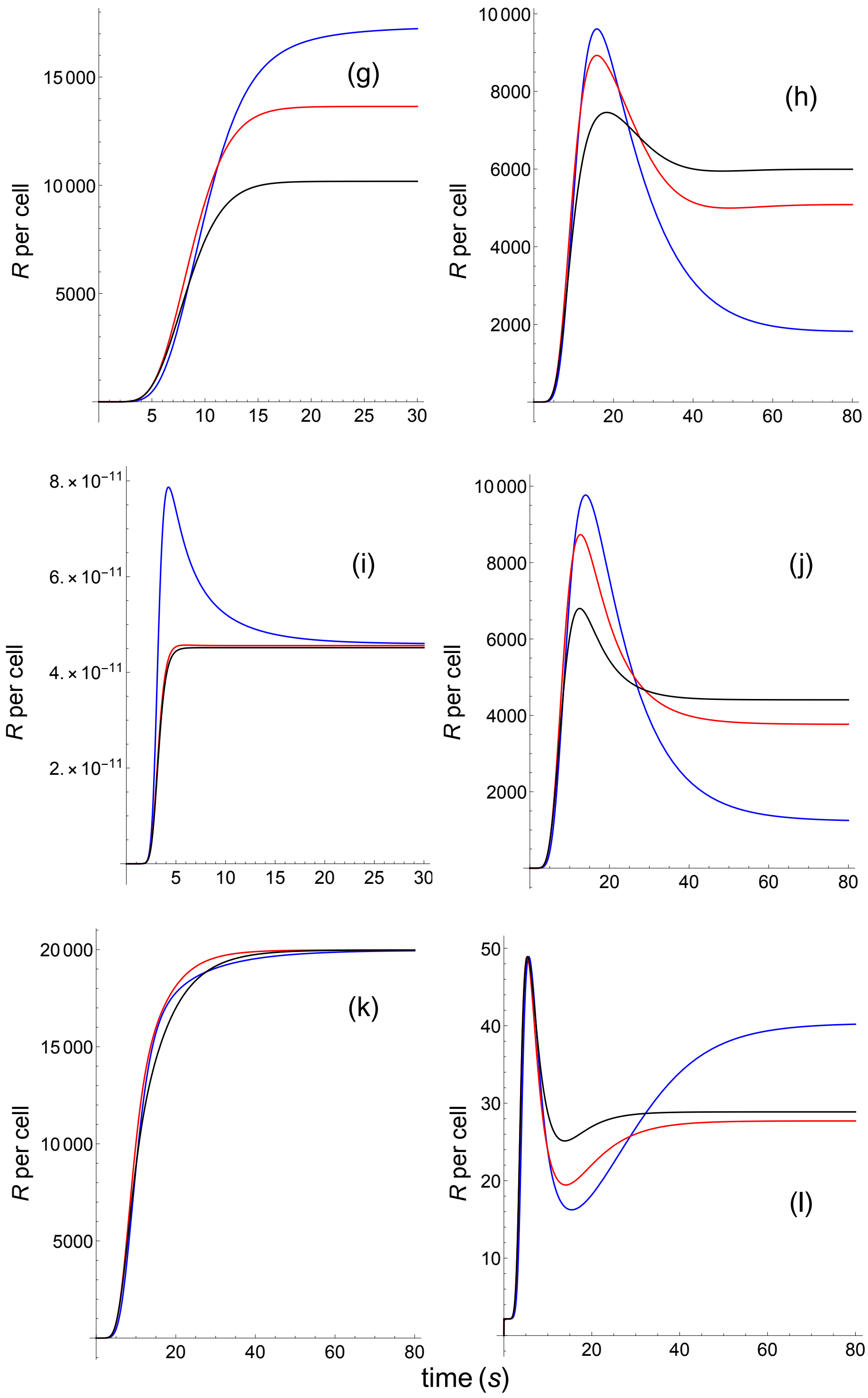

Figure 2BS(+): Dependence of the values of R on time for three systems with equal affinity (A) but different values of k_{on} (s^{-1}) and k_{off} (s^{-1}). Panel models: **(g)** kpr with stabilizing activation chain; **(h)** kpr with limited and sustained signalling; **(i)** kpr with negative feedback and limited signalling; **(j)** kpr with stabilizing activation chain and limited signalling; **(k)** kpr with stabilizing activation chain and sustained signalling; **(l)** kpr with limited signalling coupled to an incoherent feed-forward loop. Other conditions as in Figure 2AS(+).

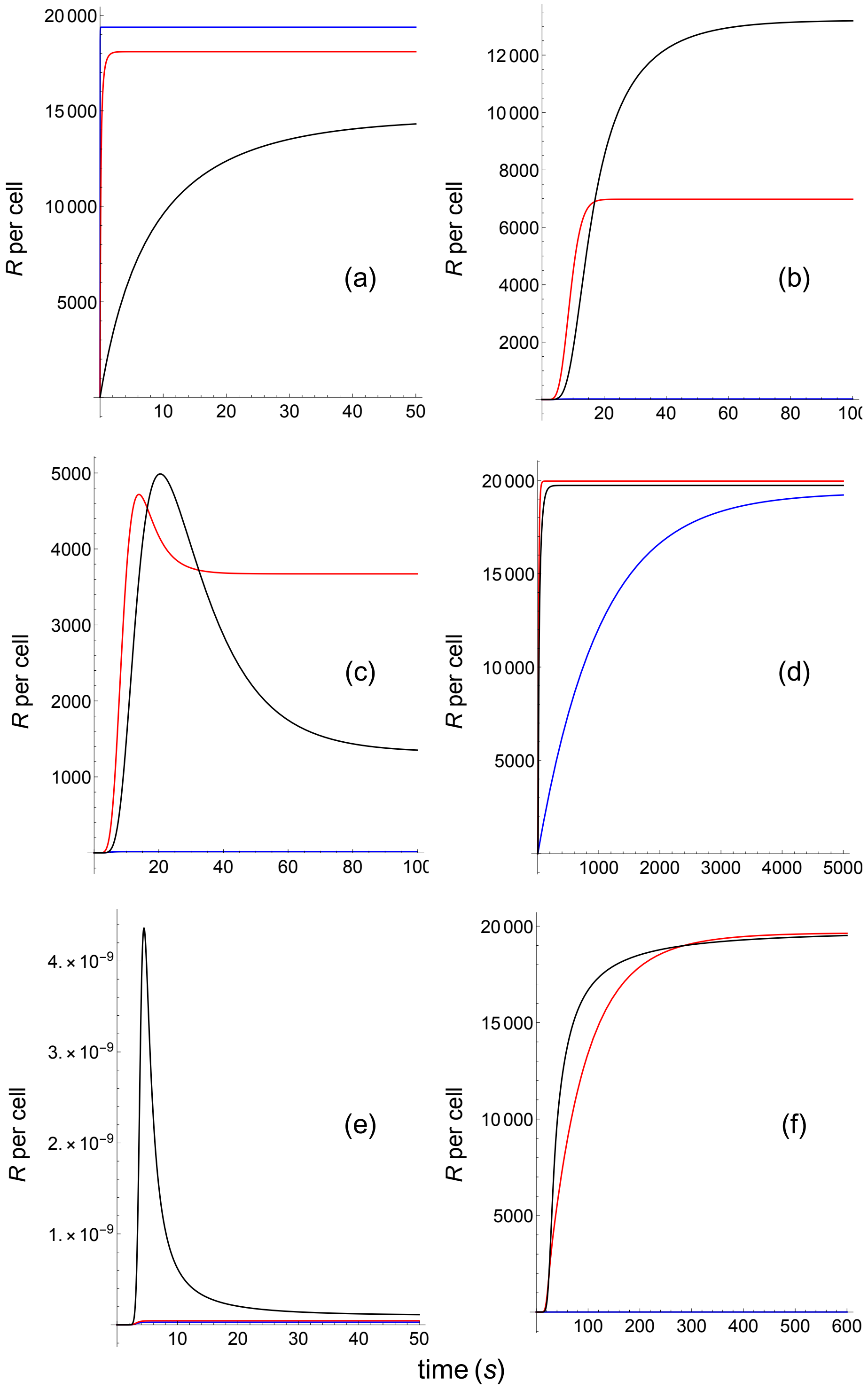

Figure 3AS(+): Dependence of the values of R on time for three systems with different affinities (A) and different values of $k_{\mathrm{on}}\,(\mathrm{s}^{-1})$ and $k_{\mathrm{off}}\,(\mathrm{s}^{-1})$ given in the form (k_{on},k_{off}): system 1: ($1000k_{\mathrm{on}}$, 1), $\tau = 1\,\mathrm{s}$, $A = 1000k_{\mathrm{on}}$ (blue); system 2: ($10k_{\mathrm{on}}$, 0.10), $\tau = 10\,\mathrm{s}$, $A = 100k_{\mathrm{on}}$ (red); system 3: ($0.1k_{\mathrm{on}}$, 0.01), $\tau = 100\,\mathrm{s}$, $A = 10k_{\mathrm{on}}$ (black). Panel models: **(a)** occupancy model; **(b)** basic kpr; **(c)** kpr with limited signalling; **(d)** kpr with sustained signalling; **(e)** kpr with negative feedback; **(f)** kpr with induced rebinding. In some cases (v.g. panel **b**, blue curve), R-values are so small that their plots are almost coincident with the x-axis. Other conditions as in Figure 2AS(+).

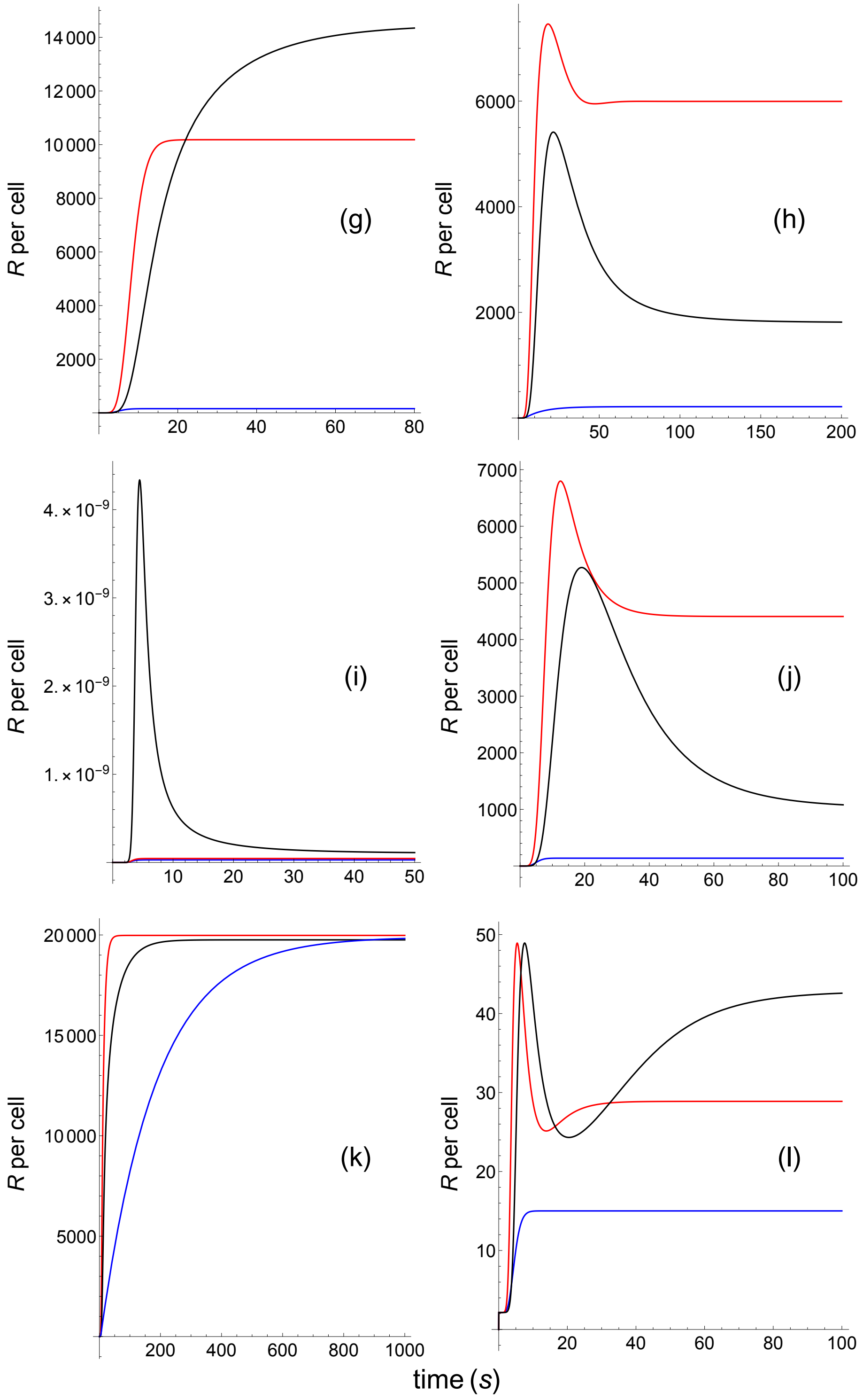

Figure 3BS(+): Dependence of the values of R on time for three systems with different affinities (A) and different values of $k_{\mathrm{on}}\,(\mathrm{s}^{-1})$ and $k_{\mathrm{off}}\,(\mathrm{s}^{-1})$. Panel models: **(g)** kpr with stabilizing activation chain; **(h)** kpr with limited and sustained signalling; **(i)** kpr with negative feedback and limited signalling; **(j)** kpr with stabilizing activation chain and limited signalling; **(k)** kpr with stabilizing activation chain and sustained signalling; **(l)** kpr with limited signalling coupled to an incoherent feed-forward loop. Other conditions as in Figures 3AS(+) and 2AS(+).

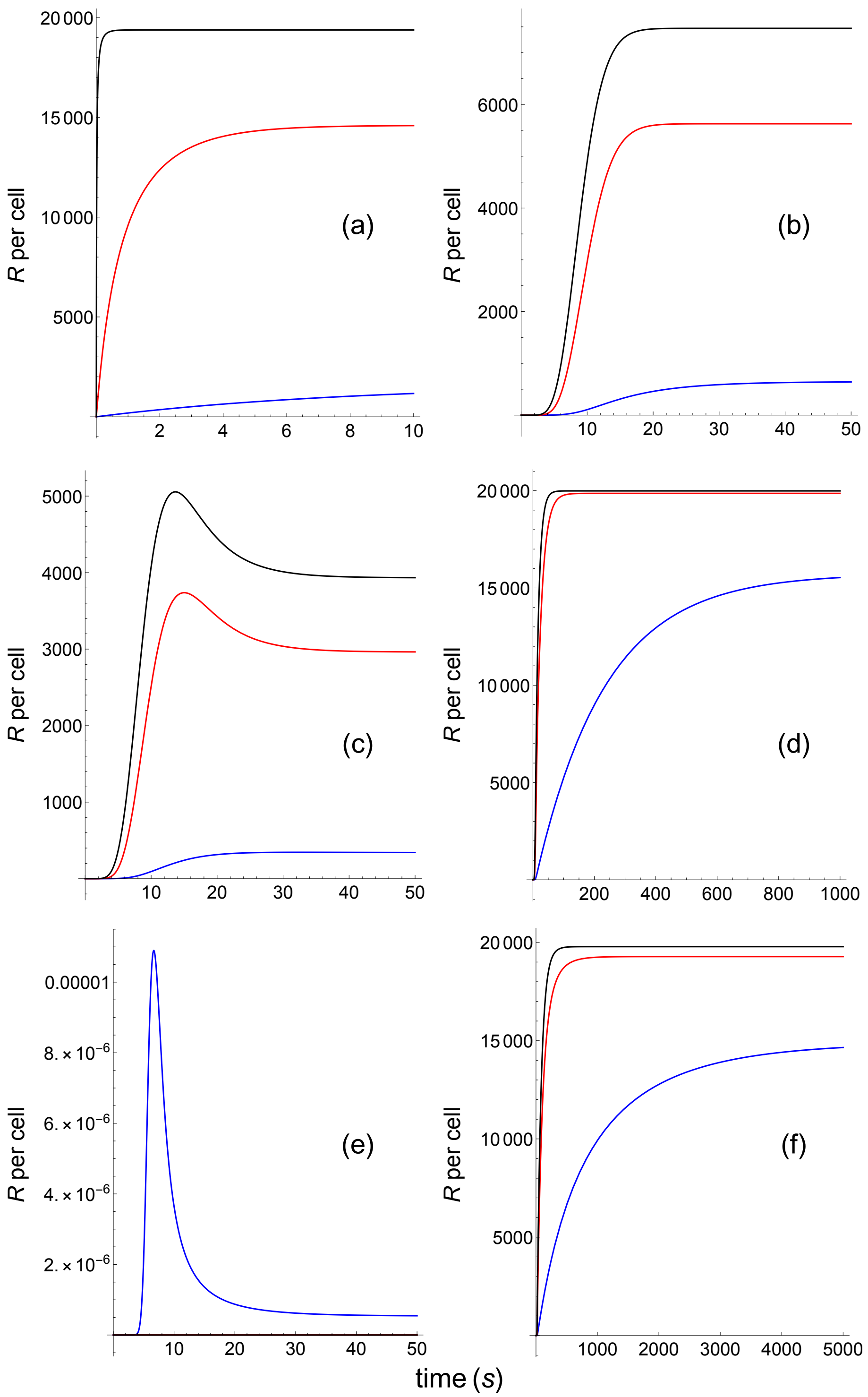

Figure 4AS(+): Dependence of the values of R on time for three systems with different affinities (A), different values of k_{on} (s^{-1}) and equal values of k_{off} (s^{-1}) given in the form (k_{on},k_{off}): system 1: ($0.01k_{\text{on}}$, 0.1), $\tau = 10\,\text{s}$, $A = 0.1k_{\text{on}}$ (blue); system 2: (k_{on}, 0.1), $\tau = 10\,\text{s}$, $A = 10k_{\text{on}}$ (red); system 3: ($100k_{\text{on}}$, 0.1), $\tau = 10\,\text{s}$, $A = 1000k_{\text{on}}$ (black). Panel models: **(a)** occupancy model; **(b)** basic kpr; **(c)** kpr with limited signalling; **(d)** kpr with sustained signalling; **(e)** kpr with negative feedback; **(f)** kpr with induced rebinding. Other conditions as in Figure 2AS(+).

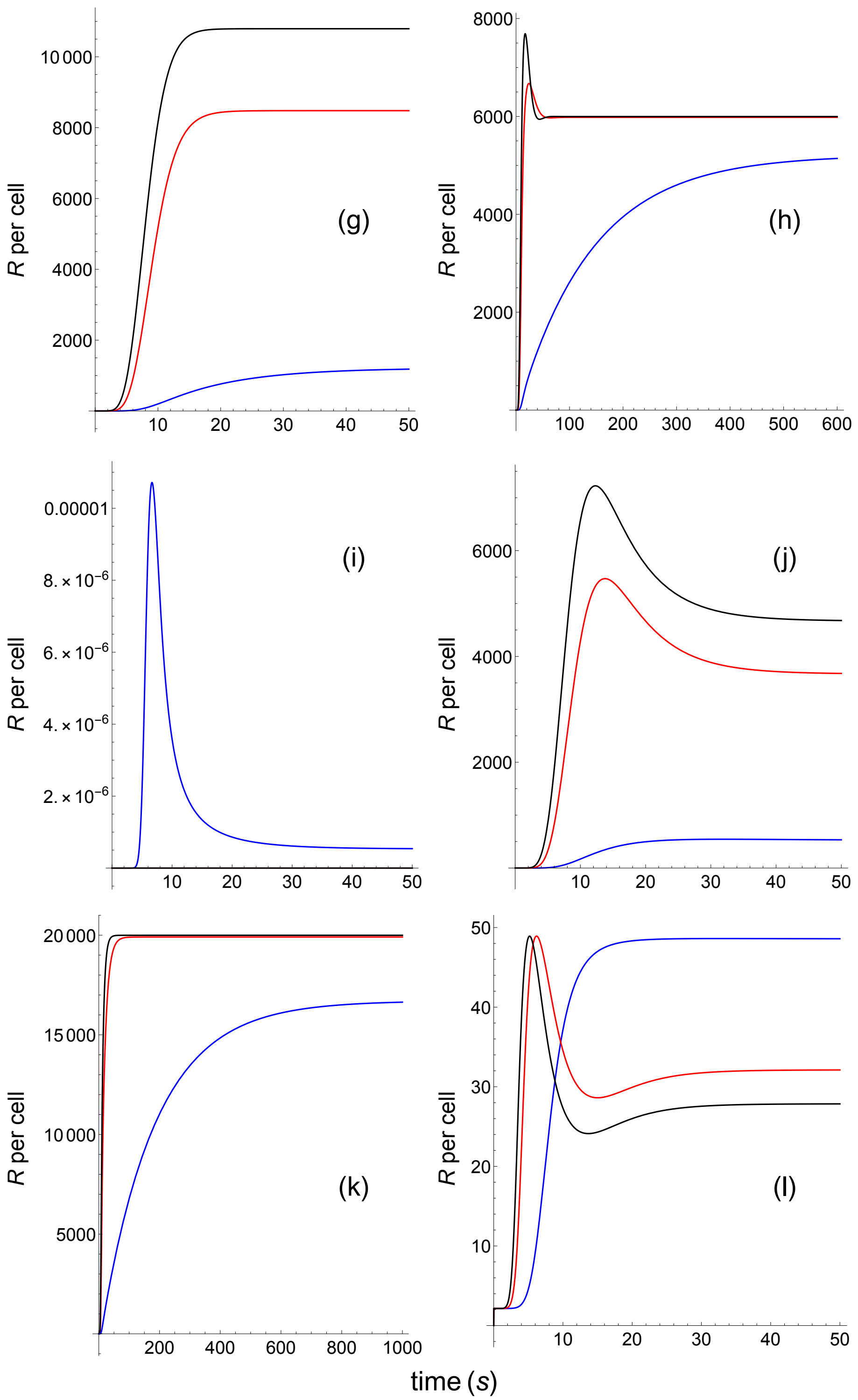

Figure 4BS(+): Dependence of the values of R on time for three systems with different affinities (A), different values of $k_{\rm on}$ ($\rm s^{-1}$) and equal values of $k_{\rm off}$ ($\rm s^{-1}$). Panel models: **(g)** kpr with stabilizing activation chain; **(h)** kpr with limited and sustained signalling; **(i)** kpr with negative feedback and limited signalling; **(j)** kpr with stabilizing activation chain and limited signalling; **(k)** kpr with stabilizing activation chain and sustained signalling; **(l)** kpr with limited signalling coupled to an incoherent feed-forward loop. Other conditions as in Figures 4AS(+) and 2AS(+).

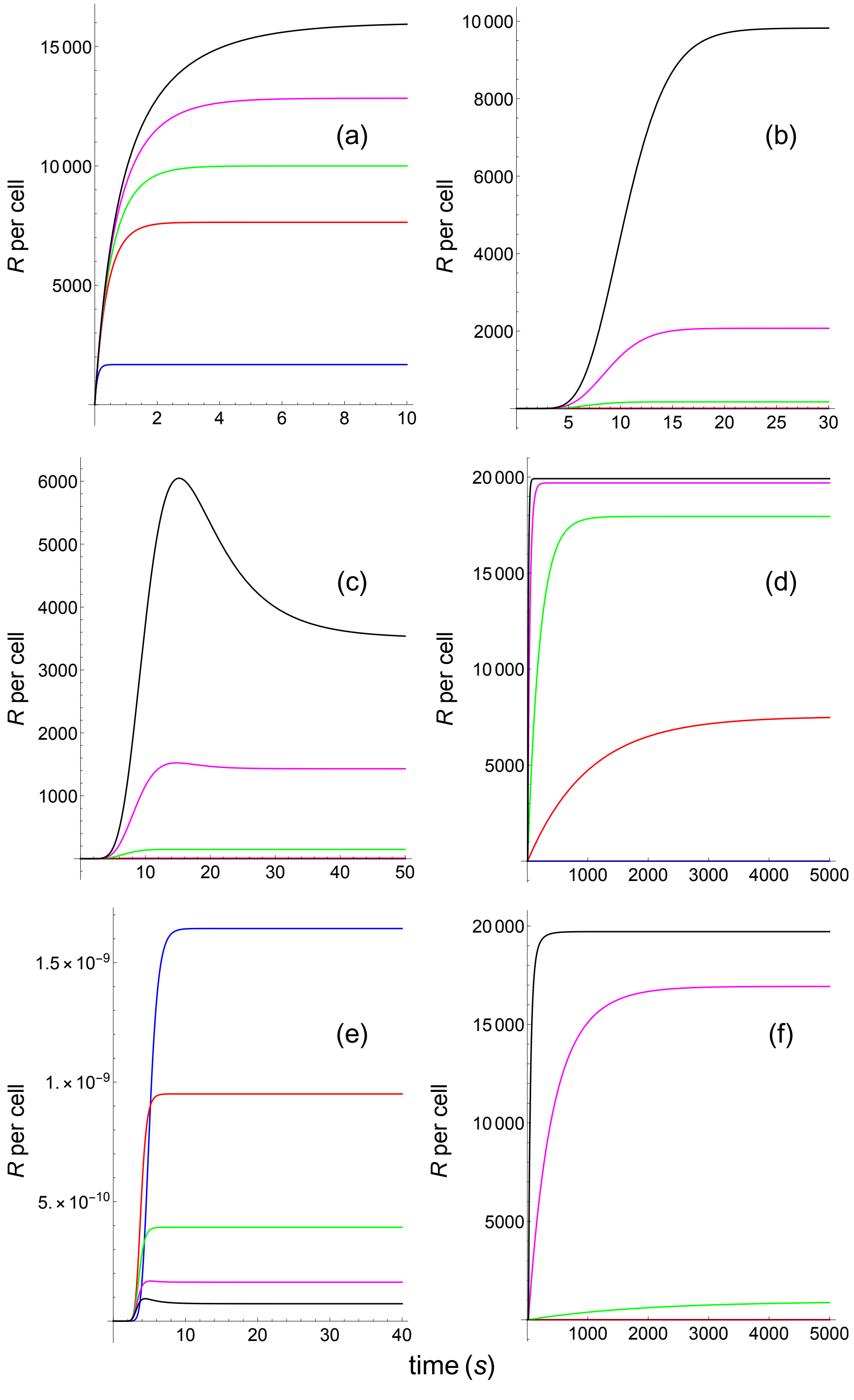

Figure 5AS(+): Dependence of the values of R on time for five systems with different affinities (A), different values of k_{off} (s^{-1}) and equal values of k_{on} (s^{-1}) given in the form (k_{on},k_{off}): system 1: (k_{on}, 10), $\tau = 0.1\,\mathrm{s}$, $A = 0.1k_{\mathrm{on}}$ (blue); system 2: (k_{on}, 1), $\tau = 1\,\mathrm{s}$, $A = k_{\mathrm{on}}$ (red); system 3: (k_{on}, 0.5), $\tau = 2\,\mathrm{s}$, $A = 2k_{\mathrm{on}}$ (green); system 4: (k_{on}, 0.2), $\tau = 5\,\mathrm{s}$, $A = 5k_{\mathrm{on}}$ (magenta); system 5: (k_{on}, 0.05), $\tau = 20\,\mathrm{s}$, $A = 20k_{\mathrm{on}}$ (black). Panel models: **(a)** occupancy model; **(b)** basic kpr; **(c)** kpr with limited signalling; **(d)** kpr with sustained signalling; **(e)** kpr with negative feedback; **(f)** kpr with induced rebinding. Other conditions as in Figure 2AS(+).

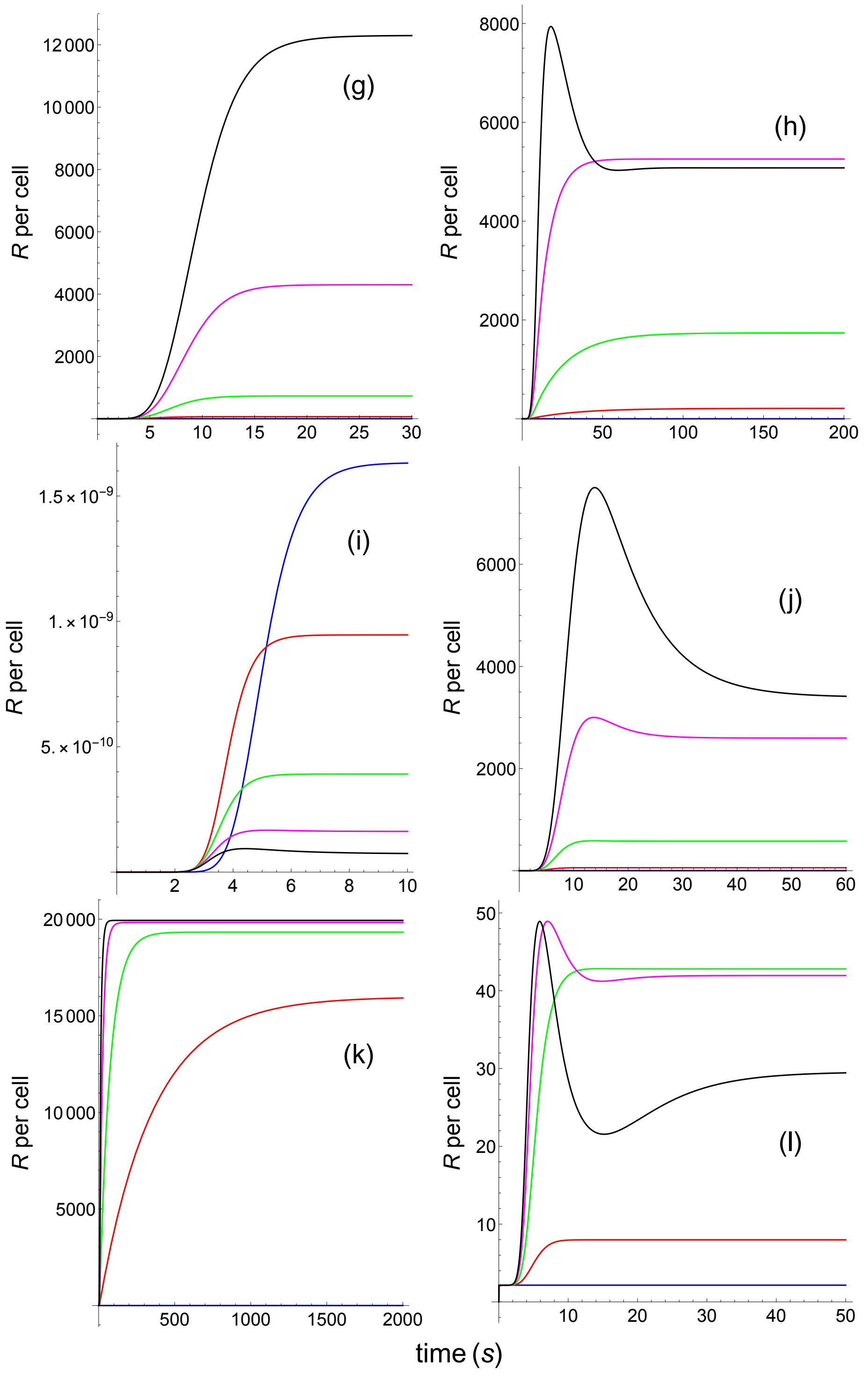

Figure 5BS(+): Dependence of the values of R on time for five systems with different affinities (A), different values of k_{off} (s^{-1}) and equal values of k_{on} (s^{-1}). Panel models: **(g)** kpr with stabilizing activation chain; **(h)** kpr with limited and sustained signalling; **(i)** kpr with negative feedback and limited signalling; **(j)** kpr with stabilizing activation chain and limited signalling; **(k)** kpr with stabilizing activation chain and sustained signalling; **(l)** kpr with limited signalling coupled to an incoherent feed-forward loop. Other conditions as in Figures 5AS(+) and 2AS(+).

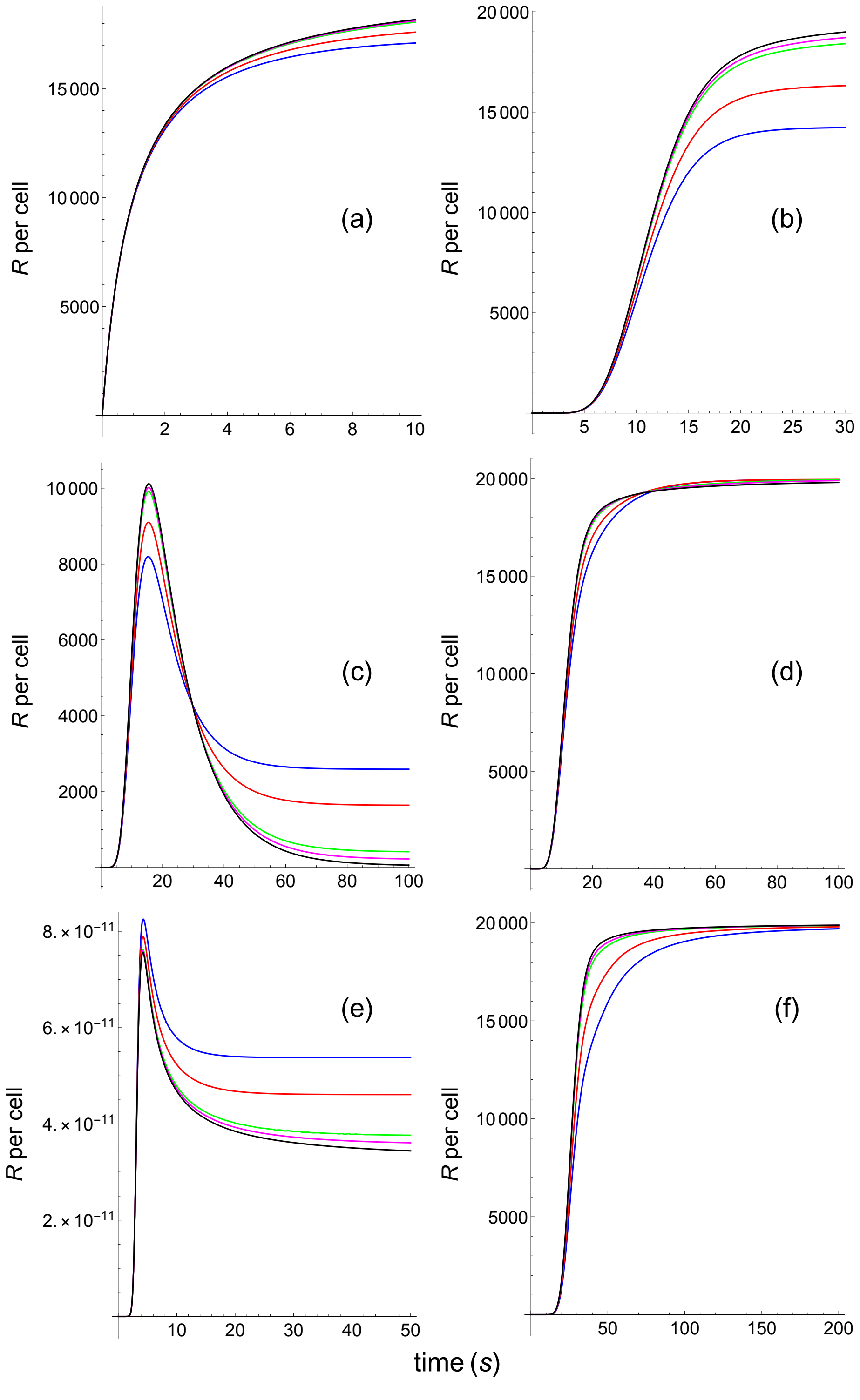

Figure 6AS(+): Dependence of the values of R on time for five systems with different affinities (A), different values of $k_{\mathrm{off}}\,(\mathrm{s}^{-1})$ and equal values of $k_{\mathrm{on}}\,(\mathrm{s}^{-1})$ given in the form (k_{on},k_{off}): system 6: (k_{on}, 0.02), $\tau = 50\,\mathrm{s}$, $A = 50k_{\mathrm{on}}$ (blue); system 7: (k_{on}, 0.01), $\tau = 100\,\mathrm{s}$, $A = 100k_{\mathrm{on}}$ (red); system 8: (k_{on}, 0.002), $\tau = 500\,\mathrm{s}$, $A = 500k_{\mathrm{on}}$ (green); system 9: (k_{on}, 0.001), $\tau = 1000\,\mathrm{s}$, $A = 1000k_{\mathrm{on}}$ (magenta); system 10: (k_{on}, 0.0001), $\tau = 10000\,\mathrm{s}$, $A = 10000k_{\mathrm{on}}$ (black). Panel models: **(a)** occupancy model; **(b)** basic kpr; **(c)** kpr with limited signalling; **(d)** kpr with sustained signalling; **(e)** kpr with negative feedback; **(f)** kpr with induced rebinding. Other conditions as in Figure 2AS(+).

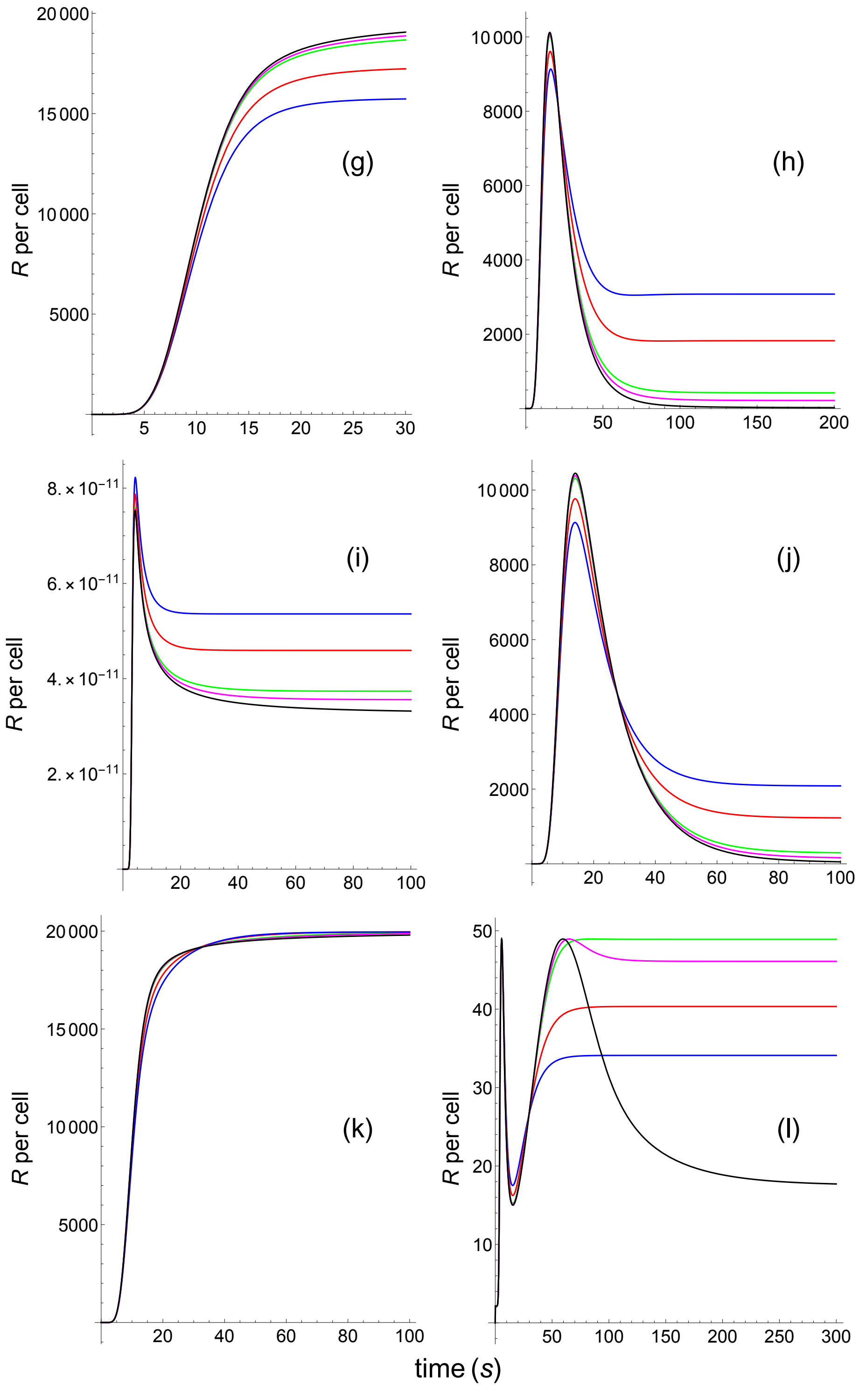

Figure 6BS(+): Dependence of the values of R on time for five systems with different affinities (A), different values of k_{off} (s^{-1}) and equal values of k_{on} (s^{-1}). Panel models: **(g)** kpr with stabilizing activation chain; **(h)** kpr with limited and sustained signalling; **(i)** kpr with negative feedback and limited signalling; **(j)** kpr with stabilizing activation chain and limited signalling; **(k)** kpr with stabilizing activation chain and sustained signalling; **(l)** kpr with limited signalling coupled to an incoherent feed-forward loop. Other conditions as in Figures 6AS(+) and 2AS(+).

References

1. Dushek O, van der Merwe, PA. An induced rebinding model of antigen discrimination. *Trends in Immunology* (2014) **35:**153-158.
